# ER-associated degradation pathway protein SEL1L plays an evolutionarily conserved role in platelet adhesion

**DOI:** 10.1172/JCI191433

**Published:** 2026-02-16

**Authors:** Anna R. Dahlgren, Francesca Careddu, Jeffrey W. Norris, Christian A. Di Buduo, Livia Stanger, Reheman Adili, Erin M. Kropp, Qing Li, Michael Holinstat, Ida Biunno, Alessandra Balduini, Fern Tablin, Jordan A. Shavit, Carrie J. Finno

**Affiliations:** 1 Department of Population Health and Reproduction, School of Veterinary Medicine, University of California Davis, Davis, California, USA.; 2 Department of Pediatrics, University of Michigan, Ann Arbor, Michigan, USA.; 3 Department of Molecular Medicine, University of Pavia, Pavia, Italy.; 4 Department of Anatomy, Physiology & Cell Biology, School of Veterinary Medicine, University of California, Davis, Davis, California, USA.; 5 Department of Pharmacology, Midwestern University, Glendale, Arizona, USA.; 6 Department of Pharmacology, University of Michigan, Ann Arbor, Michigan, USA.; 7 Blood Works Northwest Research Institute, Seattle, Washington, USA.; 8 Department of Internal Medicine, Hematology Oncology Division, University of Michigan, Ann Arbor, Michigan, USA.; 9 VA Ann Arbor Healthcare System, Medicine Service (111), Ann Arbor, Michigan, USA.; 10 Department of Cell and Developmental Biology and; 11 Department of Human Genetics, University of Michigan, Ann Arbor, Michigan, USA.

**Keywords:** Genetics, Hematology, Vascular biology, Coagulation, Genetic diseases, Platelets

## Abstract

SEL1L is a well-known protein in the ER-associated degradation (ERAD) pathway. While it is known to be expressed in platelets, SEL1L has never been shown to play an active role. Here, we present evidence that SEL1L regulates platelet function. We first identified SEL1L through the study of Atypical Equine Thrombasthenia (AET), an autosomal recessive platelet disorder found in thoroughbred horses. A missense variant in *SEL1L* (c.1810A>G p.Ile604Val) was found in AET-affected horses, which we show is associated with decreased protein expression. SEL1L is intracellular in equine platelets and localizes to the surface upon activation with thrombin. Platelets from homozygous horses exhibited substantially decreased spreading on immobilized collagen. Human megakaryocytes were found to have 2 SEL1L protein isoforms that increase in expression during megakaryopoiesis, although only 1 isoform was delivered to mature platelets. Studies using inducible mouse and constitutive zebrafish KOs demonstrated that SEL1L is necessary for efficient platelet or thrombocyte (fish equivalent) adhesion to sites of endothelial injury. These data reveal a previously undescribed and conserved role for the ERAD pathway in the etiology of AET and platelet function, and GWAS data suggest that it may play a role in human platelet disorders as well.

## Abstract 2

Using a multi-species approach, SEL1L was determined to have a role in platelet function, specifically in helping platelets properly adhere to sites of injury.

## Graphical abstract

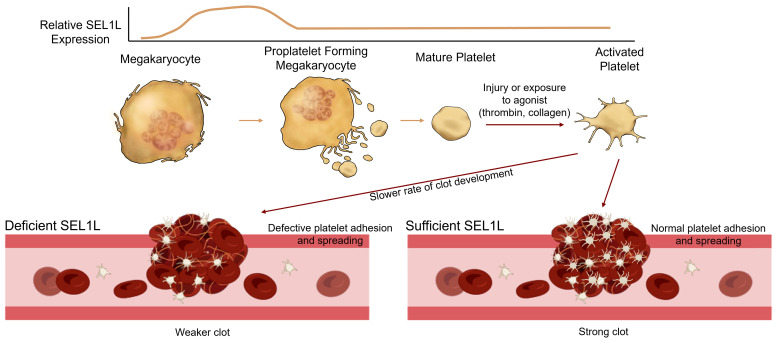

## Introduction

Genetic diseases discovered in nonhuman animals offer the opportunity to reveal genes involved in human disease. An inherited platelet dysfunction known as atypical equine thrombasthenia (AET) has been identified in thoroughbred (TB) horses that is unlike other known genetic bleeding disorders. AET results in reduced platelet response to the physiological agonist thrombin, leading to abnormal bleeding after vascular injury (1, 2). Furthermore, this inhibition can result in prolonged bleeding and, in 1 reported index case, epistaxis during racing (1). One prevalence study has been performed and demonstrated that on a single breeding farm, 1 in every 150 TBs was affected (3); however, the frequency of the disease in the general TB population is not known. Pedigrees indicate that the disorder has a recessive mode of inheritance (2).

Coagulation factors have been found to be within normal limits, and platelet counts are normal, suggesting that AET is caused by platelet dysfunction (1). Platelet aggregometry studies, using physiological agonists, demonstrate that AET platelets respond normally to ADP, only respond to collagen with supplementary calcium, and have a minimal response to the most potent agonist, thrombin (1, 2). Further research has identified biochemical differences in the AET platelet response to thrombin. After activation, the membrane phosphoinositol pathway is upregulated, resulting in the eventual phosphorylation of AKT and release of platelet α-granule contents. AET platelets have disruptions in the phosphoinositol activation pathway, as evidenced by decreased levels of phosphatidylinositol-3,4,5-trisphosphate 5-phosphatase 1 (SHIP1) in the platelet membrane, as well as the increased association of PIK3C2B, a PI3K subunit within the membrane. These 2 changes likely impact the phosphorylation of AKT and downstream α-granule release (4). α-Granules containing factor V (FV) are critical for the acceleration of thrombin activation. AET platelet α-granules contain similar levels of FV as control platelets; however, the amount released is decreased (Supplemental Figure 1; supplemental material available online with this article; https://doi.org/10.1172/JCI191433DS1) (4). This suggests a failing in the exocytosis of α-granules by activated platelets.

Here, we report candidate and genome-wide genetic analysis of AET that identifies a potentially causative missense variant in *SEL1L*. We follow this with functional confirmation in multiple model systems, including horse, human, mouse, and zebrafish. This work confirms a role of SEL1L in platelet function, which could be the underlying cause of some unknown human bleeding disorders.

## Results

### Platelet fibrinogen binding is defective in AET.

**Figure 1:**
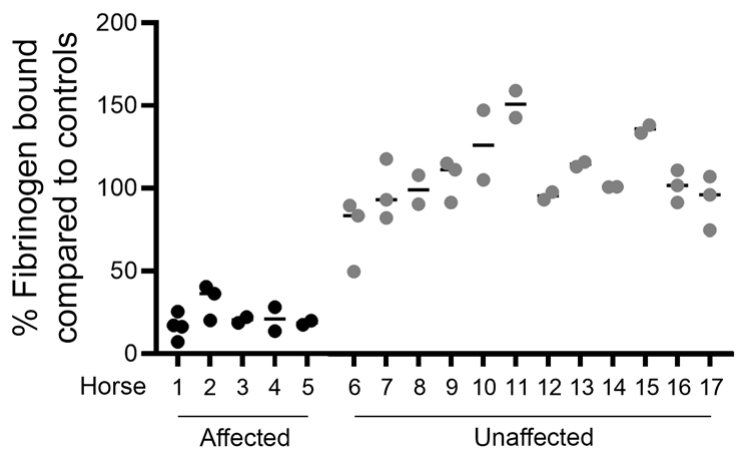
Relative fibrinogen percentage bound by platelets from affected and unaffected horses. Percent fluorescent fibrinogen bound by thrombin-activated platelets for affected (1–5) and unaffected horses (6–17) compared with the average of 3 control horses. Each horse was studied in duplicate or triplicate. The median for each horse is indicated by the horizontal bar. The affected all have an average percent fibrinogen bound under 35%.

Five TBs were identified as being AET affected in previous studies (1–3). They had 16.6%–32.4% fibrinogen bound by platelets compared with controls (Figure 1). A large degree of intra- and interindividual variability was identified. All unaffected TBs had >90% fibrinogen bound by platelets compared with controls, except for horse 6, whose dam (horse 3) and full sibling (horse 4) were AET affected.

### Candidate gene exclusion.

The 5 affected and 12 control horses were whole-genome sequenced. All affected horses were confirmed not to have the known equine variants associated with Glanzmann thrombasthenia (5–7). Across the 16 candidate loci, 8,364 variants were identified (Supplemental Table 2), but none were significantly associated with AET individuals (*P* > 0.05) when either a Bonferroni-corrected *P* value or a more generous 10% FDR was calculated.

### Whole-genome association identifies variants of interest.

Variant callers Freebayes and Delly identified 18,367,554 and 180,965 variants across the genome, respectively. A *P* < 0.0001 cutoff, which allowed half of the controls to be heterozygous, yielded 3,769 Freebayes (Supplemental Figure 2) and 10 Delly variants. Approximately 70% (2,626 variants) of the Freebayes variants were in an approximately 6 Mb region on chromosome 24 (21–27 Mb; Supplemental Figure 2). All 10 of the Delly variants were in this same region.

Five Freebayes variants were predicted to have a moderate effect on protein function. Only 1 was in a gene known to be expressed in platelets and to interact with other platelet proteins (8) (Supplemental Table 3). This was a missense variant in exon 18 of the *suppressor/enhancer of lin-12-like* gene (*SEL1L* c.1810A>G p.Ile604Val; *P* = 7.63 × 10^−6^). Two Delly variants were in or near a gene expressed in platelets. One was a deletion in an uncharacterized long noncoding RNA (lncRNA) upstream of *SEL1L*, according to TransMap (genome.ucsc.edu; *AL355838.1:g.26447375_26448962del*; *P* = 7.63 × 10^−6^), and the other was an intronic deletion in *VIPAS39* (g.22685398-22685470del; *P* = 3.74 × 10^−5^).

### RT-PCR confirms gene expression in horse platelets.

To confirm that *SEL1L*, the upstream lncRNA, and *VIPAS39* were expressed in equine platelets, end-point PCR was performed with cDNA from unaffected platelets and testicular tissue as a positive control. The observed amplicons demonstrated that *SEL1L* and *VIPAS39* are expressed in equine platelets, but not the upstream lncRNA (Supplemental Figure 3).

### Population study confirms association.

**Figure 2:**
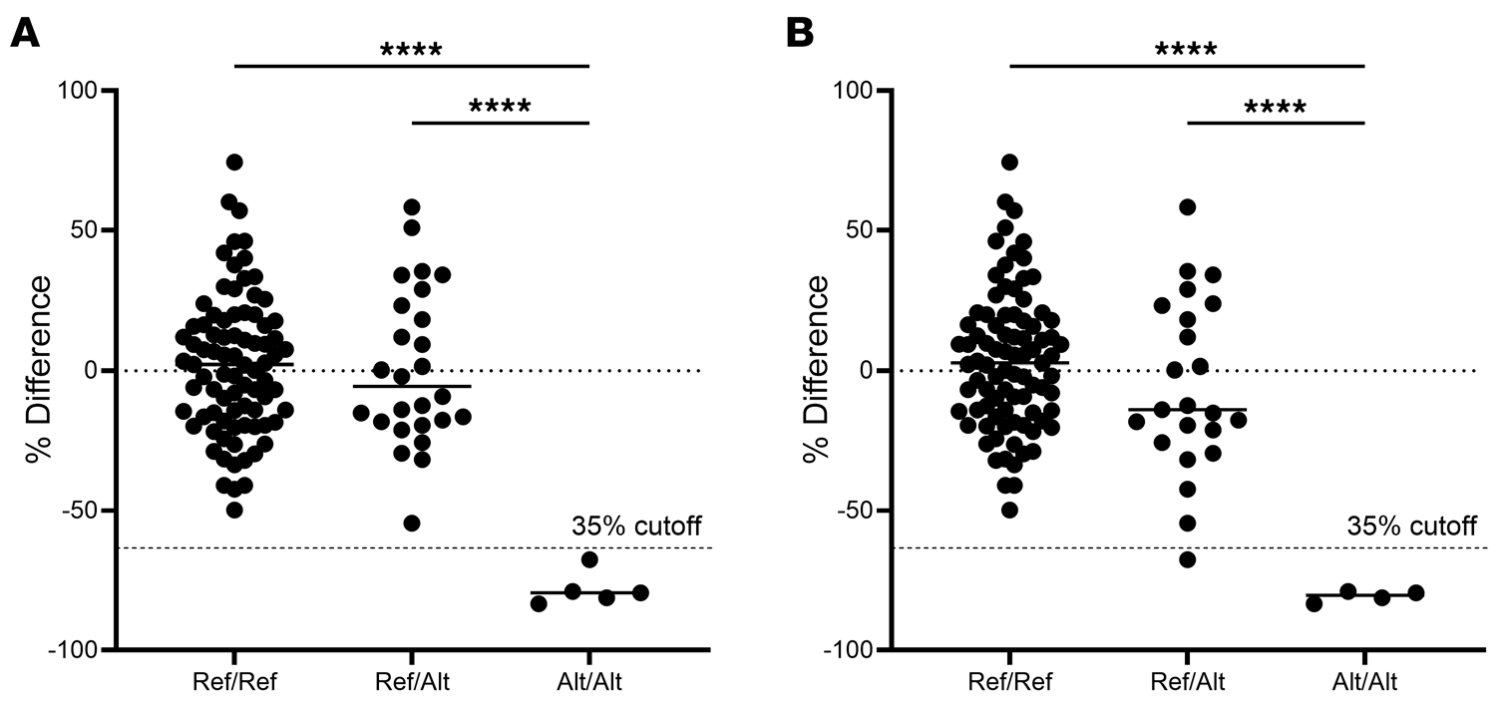
Genotypes of the larger population of phenotyped horses. Genotypes of the larger population of 114 phenotyped TBs for (**A**) *SEL1L* c.1810A>G p.Ile604Val and (**B**) *VIPAS39:g.22685398_22685470del*. The *y* axis is percent difference in fibrinogen binding compared with control horses. The 35% cutoff line characterizing affected horses is indicated by a dashed line. ****P < 0.0001 by 1-way ANOVA.

A larger population of 114 TBs, including some of the sequenced horses, that had previously been phenotyped (2, 3) were genotyped for the *SEL1L* and *VIPAS39* variants. All affected horses were homozygous for the *SEL1L* variant (i.e., alternate allele; Alt/Alt). Platelet binding to fibrinogen was similar between the heterozygous (Ref/Alt) and homozygous WT (Ref/Ref) horses but was significantly different from the Alt/Alt horses (Figure 2A; *P* = 2.0 × 10^−9^). For the *VIPAS39* variant, there was a significant difference in fibrinogen binding between the Alt/Alt horses and the other 2 genotypes (Figure 2B; *P* = 2.0 × 10^−9^), but 1 horse below the 35% cutoff was heterozygous.

### Candidate gene expression in platelets is unaffected by variants.

To determine if either variant affected mRNA expression of their respective genes, RT-qPCR was performed on platelet mRNA. Neither *SEL1L* (*P* = 0.74) nor *VIPAS39* (*P* = 0.64) showed a change in expression associated with the variants of interest (Supplemental Figure 4).

Further evaluation of the intronic *VIPAS39* deletion locus in publicly available data from the Functional Annotation of the Animal Genomes project (9) did not reveal any known regulatory elements, suggesting it does not play a role in *VIPAS39* function. Additionally, the AET phenotype is nothing like the Arthrogryposis Renal Dysfunction Cholestasis Syndrome phenotype seen in humans that have known *VIPAS39* deleterious variants (10–12). Thus, the intronic *VIPAS39* deletion was excluded from further study.

### Flow cytometry identifies changes in protein expression.

**Figure 3:**
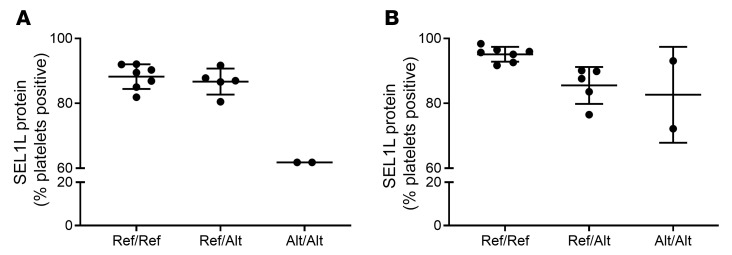
SEL1L protein expression in resting and activated equine platelets. SEL1L protein expression from flow cytometry in (**A**) resting permeabilized platelets and (**B**) on the surface of thrombin-activated platelets. The median for each horse and the SD are shown. *n* = 7 Ref/Ref, 6 Ref/Alt, and 2 Alt/Alt. The percent platelets that were positive for SEL1L was decreased in the Alt/Alt horses compared with the Ref/Ref horses in both resting and activated platelets.

Since point mutations can affect protein expression without affecting mRNA levels (13), we investigated SEL1L protein expression using flow cytometry. There were substantially fewer platelets with intracellular SEL1L in horses that were homozygous for the *SEL1L* variant compared with Ref/Ref (Figure 3A), though the low sample size of Alt/Alt horses precluded statistical analysis. There was also a nonsignificant decrease in platelets with intracellular SEL1L between Ref/Ref and Ref/Alt horses (*P* = 0.08). We next questioned whether SEL1L might play a role in platelet interactions following activation. Equine platelets were activated with thrombin, which has been shown to be an aberrant pathway in AET-affected horses (2, 4). We found that SEL1L relocated to the platelet surface upon activation (Figure 3B), and there was a trend of fewer platelets expressing surface SEL1L in AET-affected horses compared with Ref/Ref. Due to the limited sample size, the Ref/Ref and Ref/Alt groups were combined and compared with the Alt/Alt horses, and there was a significant difference (*P* = 0.005).

### SEL1L localizes to the surface upon activation and is involved in platelet spreading.

**Figure 4:**
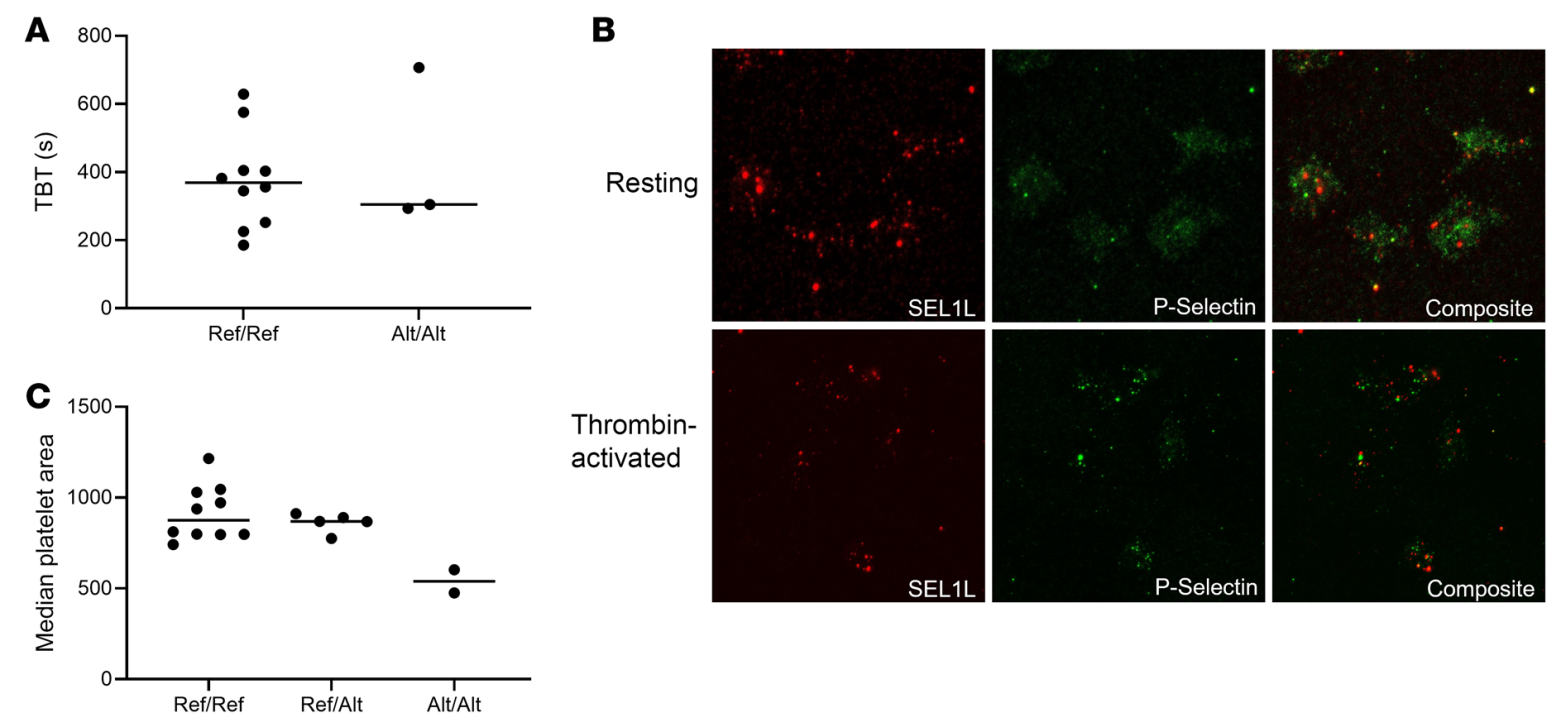
Clinical phenotyping and analysis of platelets from affected horses. (**A**) TBTs using horses that were Ref/Ref or Alt/Alt for the *SEL1L* c.1810A>G p.Ile604Val variant found no significant difference between genotypes by 2-tailed Student’s *t* test. *n* = 10 Ref/Ref and 3 Alt/Alt. (**B**) SEL1L (red) localization in relation to P selectin (green) in (top) resting permeabilized platelets and (bottom) thrombin-activated platelet surface of horses that are Ref/Ref for *SEL1L* c.1810A>G p.Ile604Val. SEL1L did not localize to the surface in resting permeabilized platelets (top) but did localize to the surface in thrombin-activated platelets (bottom). *n* = 2. Original magnification, ×100. (**C**) Collagen spreading assay comparing median platelet area to genotype. There was a substantial difference between homozygous Ref and Alt genotypes. *n* = 11 Ref/Ref, 5 Ref/Alt, and 2 Alt/Alt.

We next tested various coagulation assays and determined whether there was an association with the *SEL1L* genotypes. This included a template bleeding time (TBT) study, standard clotting assays, and platelet count. No significant difference in TBT between Ref/Ref and Alt/Alt horses (*P* = 0.94; Figure 4A) was identified. We also found no differences in plasma clotting assays, fibrinogen levels, or platelet count based on *SEL1L* genotype (Supplemental Figure 5).

Our flow cytometry data suggest that SEL1L translocates to the platelet surface upon thrombin activation. We therefore evaluated whether it was localized to the α-granule. The membranes of these secretory organelles are incorporated into the platelet membrane during exocytosis (14). Permeabilized resting platelets and thrombin-activated platelets from Ref/Ref and Alt/Alt TBs were fluorescently stained for P selectin, a marker of α-granules, and SEL1L. The fluorescent markers of these 2 proteins did not overlap consistently, indicating that SEL1L does not localize to the α-granule in either WT or homozygous mutant platelets (Figure 4B and Supplemental Figure 6). However, we saw evidence that SEL1L still localizes to the surface upon thrombin activation in both genotypes.

Lastly, a collagen spreading assay was used to investigate the role of SEL1L in platelet function. First, we confirmed that there was no difference in mean platelet volume between genotypes (*P* = 0.54; Supplemental Figure 7). The average area the activated platelets spread was then determined for each horse. A substantial decrease in median platelet area was found between Ref/Ref and Alt/Alt horses (Figure 4C).

### SEL1L is expressed by megakaryocytes and delivered to platelets.

To elucidate the mechanism of action of SEL1L in platelets, differentiating human megakaryocytes were used. During megakaryopoiesis, proplatelet-forming megakaryocytes develop nascent platelets at their terminal ends, while boosting gene expression to produce proteins to be delivered to platelets (15).

**Figure 5:**
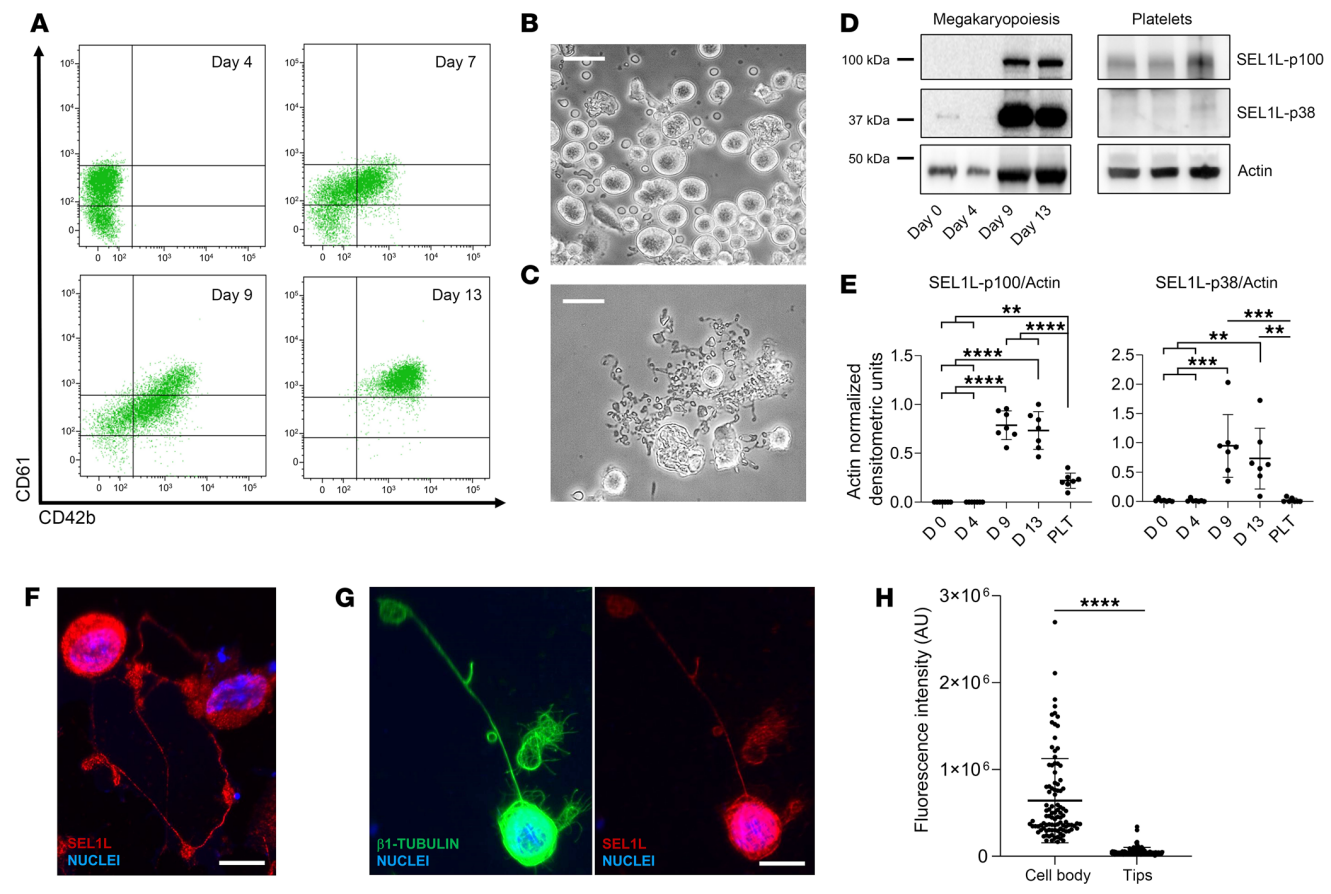
SEL1L is expressed in human cord blood megakaryocytes and platelets. (**A**) Flow cytometry analyses of human megakaryocytic surface markers from differentiated cord blood. CD61^hi^CD42b^hi^ megakaryocytes are observed after 2 weeks of differentiation. (**B**) Phase contrast microscopy of mature human megakaryocytes and (**C**) proplatelets. Scale bars: 50 μm. (**D**) Representative Western blot analysis of SEL1L during megakaryopoiesis and in platelets from peripheral blood. (**E**) Densitometric analyses of SEL1L isoforms (*n* = 7, results are presented as mean ± SD). **P < 0.01, ***P < 0.001, and ****P < 0.0001 by 1-way ANOVA with post hoc pairwise comparisons. (**F** and **G**) Immunofluorescence microscopy of megakaryocytes extending proplatelets. Scale bars: 30 μm. (**H**) Analysis of SEL1L fluorescence intensity in the megakaryocytic cell body and proplatelet tips. ****P < 0.0001 by 2-tailed Student’s *t* test.

Proplatelet-forming megakaryocytes were derived from human adult and cord blood CD34^+^ hematopoietic stem and progenitor cells (16, 17). During differentiation of cord blood, a progressive increase in the number of cells expressing lineage-specific markers was observed (Figure 5A and Supplemental Figure 8A), paralleled by cell enlargement (Figure 5B) and elongation of branched proplatelets (Figure 5C and Supplemental Figure 8B). Spontaneous platelet formation from megakaryocytes occurred on day 13 of differentiation when mature platelets were assembled at the terminal ends of the proplatelet branches. Parallel analysis of cell lysates of these megakaryocytes on different days of differentiation showed a significant increase in the expression of SEL1L during both adult and fetal megakaryopoiesis, consisting of 2 variants: SEL1L-p100kDa and SEL1L-p38kDa (Figure 5, D and E, and Supplemental Figure 8, C and D), indicating that different isoforms of SEL1L are present and regulated during megakaryopoiesis, regardless of the source of the hematopoietic progenitors.

While the SEL1L-p100kDa variant remains present in peripheral blood–derived platelets, the level of SEL1L-p38kDa decreased to undetectable levels, suggesting that the isoforms are differentially delivered and mature platelets have different requirements compared with their progenitors (Figure 5, D and E). Accordingly, immunofluorescence staining of SEL1L in proplatelet-forming megakaryocytes demonstrated that the protein is mainly localized in the cell body rather than the proplatelet tips (Figure 5, F–H). These data demonstrate that SEL1L expression is prominent during megakaryocyte differentiation, but only 1 isoform appears to be distributed to mature platelets.

We examined RNA-seq results from different stages of megakaryocyte development in mouse (18, 19) to corroborate the human data. *SEL1L* is expressed throughout murine megakaryocyte development (Supplemental Figure 9). *SEL1L* is also expressed at similarly low levels in both mouse and human platelets (Supplemental Figure 9). Overall, SEL1L is expressed throughout mammalian megakaryocyte development and passed on to platelets in both mouse and human. Using RT-PCR, the longer isoform, *SEL1L-A* (20)*,* was found to be expressed in all species’ platelets/thrombocytes (Supplemental Figure 10).

### Loss of SEL1L affects platelet adhesion in response to endothelial injury in vivo.

**Figure 6:**
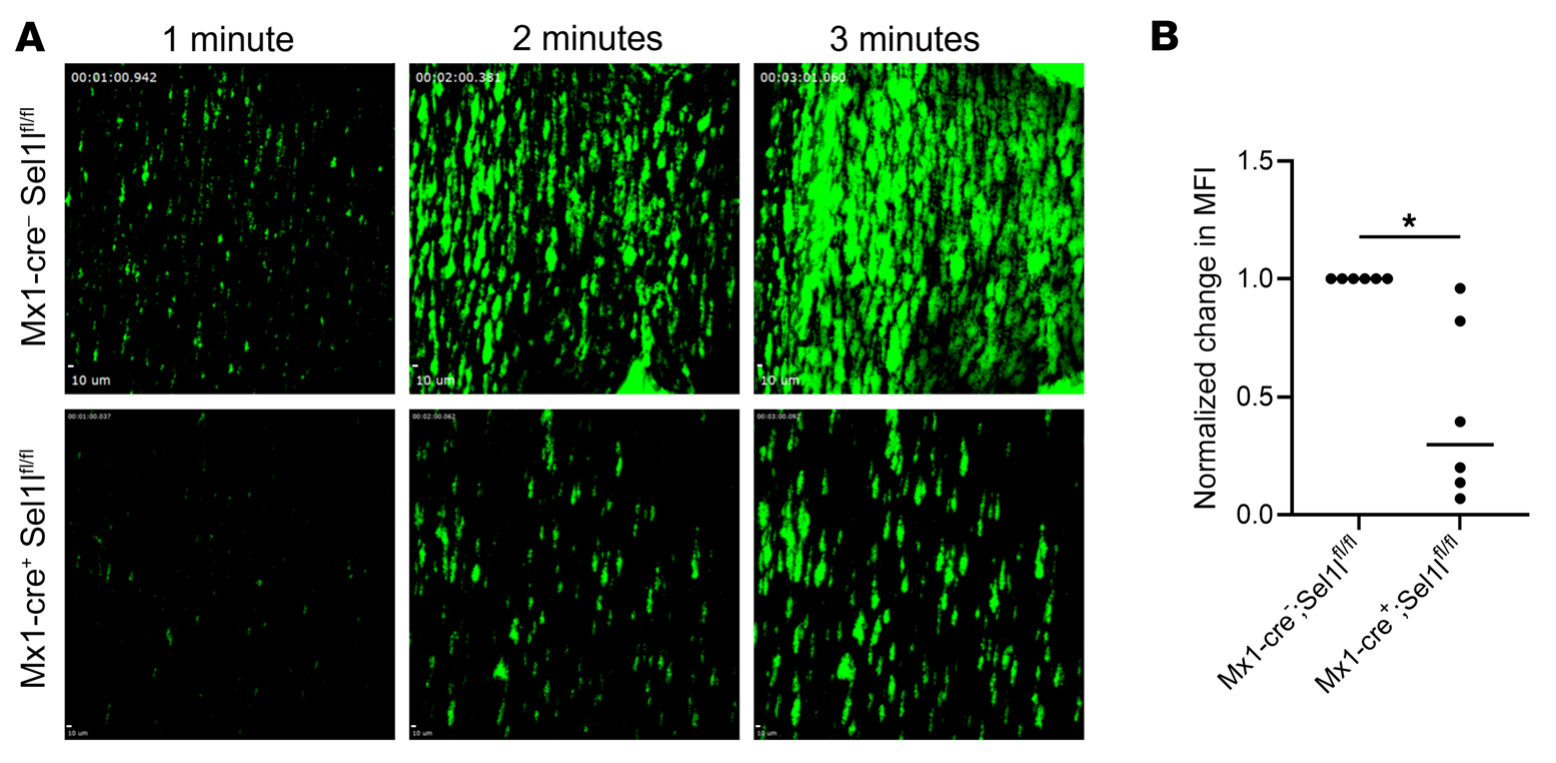
Reduced adherence to collagen in *Mx1-cre Sel1l^fl/fl^* platelets under flow. (**A**) Representative images of murine platelets (green) adhering to a collagen-coated chamber after perfusion of whole blood from both a control *Mx1-cre*^–^ *Sel1l^fl/fl^* and a knockdown *Mx1-cre^+^ Sel1l^fl/fl^* mouse. Scale bars: 10 μm. (**B**) Quantification of the change in MFI between the beginning and end of the whole blood perfusion across collagen was normalized to the control for each pair. *n* = 6 cre^+^/cre^–^ sibling pairs. *P < 0.05 by Wilcoxon’s matched-pairs signed-rank test; a significant decrease in MFI change was found between control and knockdown groups.

**Figure 7:**
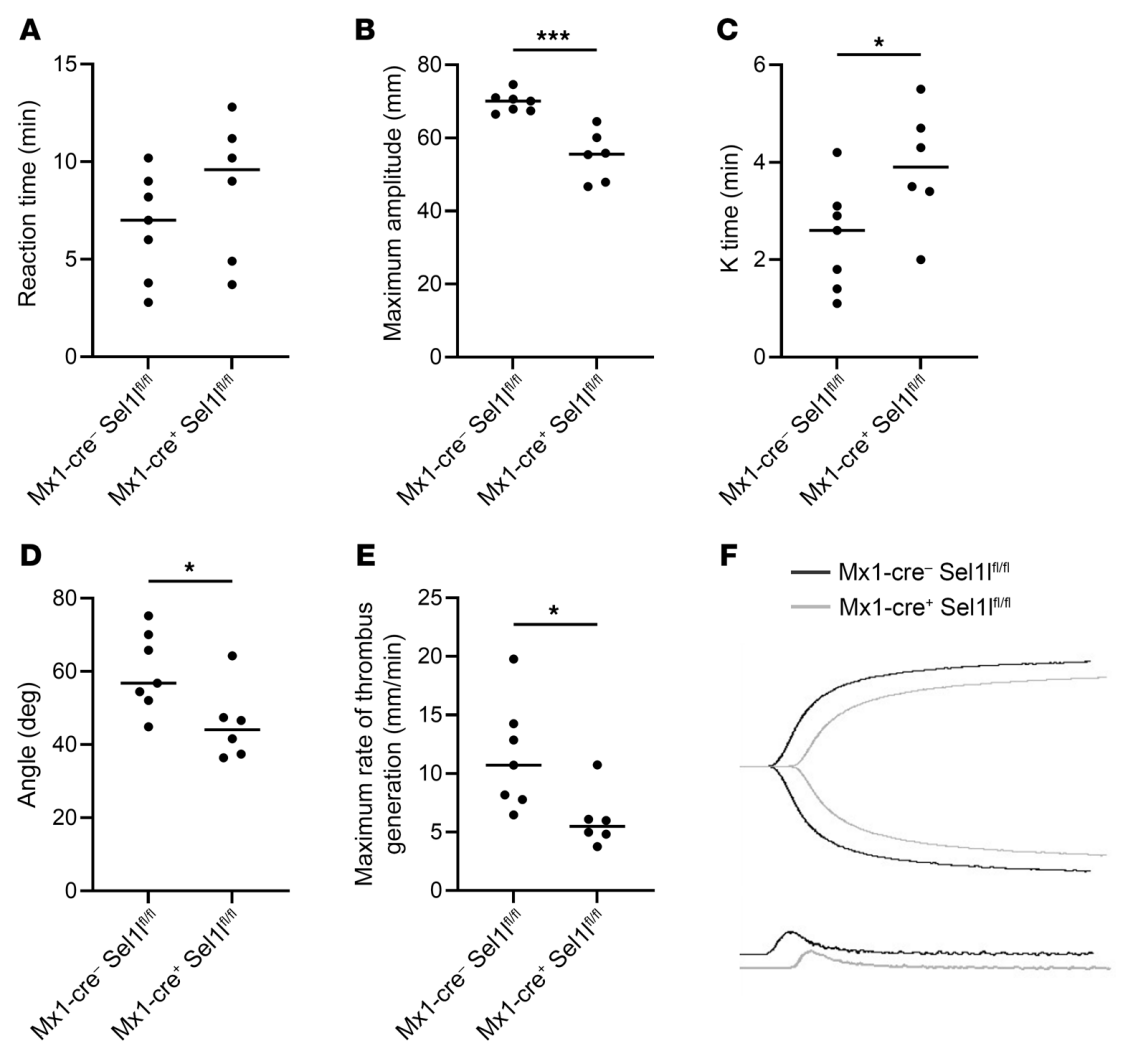
Altered TEG in *Sel1l* mutant mice. TEG was performed on control *Mx1-cre*^−^ *Sel1l^fl/fl^* (*n* = 7) and knockdown *Mx1-cre^+^ Sel1l^fl/fl^* (*n* = 6) mice. The results showed (**A**) no significant difference in reaction time, (**B**) a significant decrease in maximum amplitude, (**C**) a significant increase in the time for the clot to reach a strength of 20 mm (K time), (**D**) a significant decrease in the rate of clot formation (α-angle), and (**E**) a significant decrease in maximal rate of thrombus generation. (**F**) Representative tracings and velocity curves show the difference between the control in black and the KO in gray. *P < 0.05 and ***P < 0.001 by 2-tailed Student’s *t* test.

Given our access to only a limited number of horses, we pursued several other model systems of SEL1L deficiency for further studies. To evaluate the impact on platelet function, we examined loss-of-function mutations in mouse and zebrafish models. *Mx1-cre^+^* *Sel1l^fl/fl^* mice were used to conditionally knock out the gene in hematopoietic tissue and downstream differentiated cells, followed by 3 assays to examine platelet function. First, a tail bleeding assay demonstrated that there was no difference in bleeding time between *Mx1-cre^+^* *Sel1l^fl/fl^* and control *Mx1-cre*^−^*Sel1l^fl/fl^* mice (*P* = 0.9; Supplemental Figure 11), which corroborates the results from the horse TBT experiment. Next, platelet binding to collagen was evaluated ex vivo. Perfusion of whole blood with fluorescently labeled platelets through a collagen-coated chamber demonstrated a significant decrease in platelet adhesion in the *Mx1-cre^+^ Sel1l^fl/fl^* mice versus control littermates (*P* = 0.03; Figure 6). Lastly, thromboelastography (TEG) demonstrated a difference in platelet function between genotypes. The reaction time showed no differences, indicating the time to clot initiation was not affected (*P* = 0.3; Figure 7A). However, the maximum amplitude (*P* = 0.0003), the K time (*P* = 0.04), the α-angle (*P* = 0.03), and the maximal rate of thrombin generation (*P* = 0.03) were all significantly different between the KO and WT mice (Figure 7, B–F), suggesting SEL1L deficiency decreases rate of clot development and strength.

**Figure 8:**
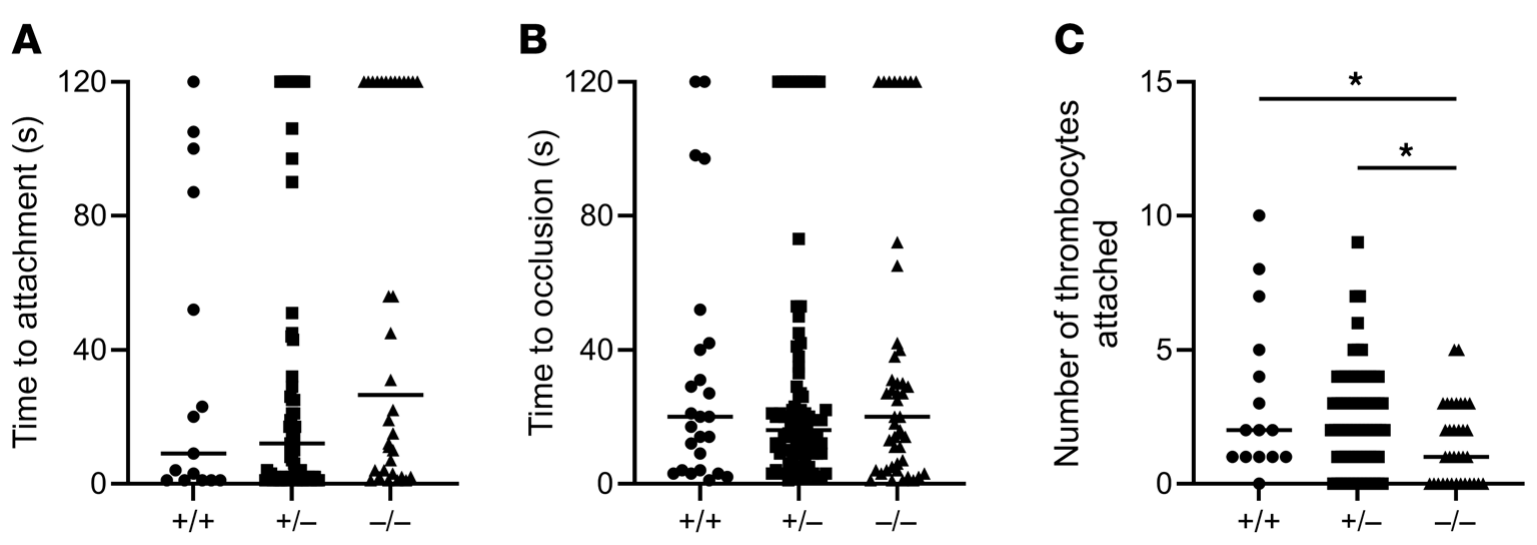
Defective thrombocyte attachment in *sel1l* mutant zebrafish. Laser-mediated arterial endothelial injury of a heterozygous incross at 5 dpf shows no difference in (**A**) time to first thrombocyte attachment (*n* = 15 +/+, 69 +/–, and 34 –/–) or (**B**) time to occlusion between WT (+/+), heterozygous (+/–), or homozygous (–/–) larvae (*n* = 25 +/+, 89 +/–, and 46 –/–), but there was a (**C**) significant decrease in the number of thrombocytes that attach to the site of injury in homozygous larvae compared with those that were WT and heterozygous (*n* = 15 +/+, 69 +/–, and 34 –/–). *P < 0.05 by Kruskal-Wallis test with multiple comparisons and an FDR correction.

We next produced a large deletion in zebrafish *sel1l* using CRISPR/Cas9 genome editing, eliminating nearly the entire coding region (Supplemental Figure 12). Laser-mediated arterial endothelial injury was performed on 5 day postfertilization (dpf) offspring of a heterozygous incross. While there was no significant difference in time to occlusion or time to first thrombocyte attachment, there was a significant difference in total number of thrombocytes that adhered (*P* = 0.02; Figure 8). There were no significant differences in total thrombocyte counts (Supplemental Figure 13).

### SEL1L is associated with bleeding phenotypes in genome-wide association analyses.

Two large GWAS accessible through PheWeb (21) were reviewed for complex phenotypes that are associated with *SEL1L*. The Michigan Genomics Initiative biobank (22), which analyzed 51.8 million imputed variants across 51,583 individuals, revealed an association between variants neighboring or within the *SEL1L* locus and 2 bleeding phenotypes. A proximal variant for which the nearest gene is *SEL1L* was the seventh most associated locus with epistaxis and throat hemorrhage (*P* = 2.5 × 10^−7^; Table 1). A SNP within *SEL1L* was also one of the top 20 loci associated with the phenotype of purpura and other hemorrhagic conditions (*P* = 6.4 × 10^−7^; Table 1). A substantially larger GWAS conducted using data from the UK Biobank (23), which includes data from 337,199 individuals and 13.7 million imputed variants, showed that a variant for which the closest adjoining gene is *SEL1L* was the 24th most associated locus with the clotting disorder/excessive bleeding phenotype category (*P* = 8.3 × 10^−11^; Table 1). These findings suggest that SEL1L may play a discernible role in bleeding phenotypes in humans.

## Discussion

This study investigated the genetic etiology of AET and discovered a role for SEL1L in platelet function that, to our knowledge, was previously unknown. AET is an inherited platelet disorder that prevents affected horses from clotting efficiently following vascular injury. Whole-genome sequencing identified a missense variant in *SEL1L* (c.1810A>G p.Ile604Val). This variant was associated with expression changes at the protein level and decreased platelet spreading on collagen. Human megakaryocyte studies determined protein localization and identified different isoforms. Mouse megakaryocyte RNA-seq corroborated the human data, with S*el1l* expression throughout megakaryocyte development. Finally, mouse and zebrafish *SEL1L*-KO models definitively identified a functional role for SEL1L in platelet function. Therefore, the missense variant is likely the cause of AET.

A limitation throughout this work was the number of horses available for study. We only identified *n* = 3 TBs that were Alt/Alt for the *SEL1L* c.1810A>G p.Ile604Val variant and that were readily accessible, since fresh platelets or horses were required for most of the equine experiments. The TBT has a high degree of both intra- and interhorse variability (24), which may have contributed to the nonsignificant results. Additionally, the high TBT seen in the index case (>120 minutes) may have been primarily due to the consumption of coagulation factors, as it was evaluated after bleeding for 3 weeks (1). We also demonstrated that, while SEL1L is an intracellular protein, it does not localize to the α-granule in resting platelets, but it does relocate to the plasma membrane upon thrombin stimulation. This indicates that SEL1L is likely localized to an organelle within the platelet that is involved in exocytosis after activation. Since SEL1L is known to localize to the ER in nucleated cells (25), we hypothesize that platelet SEL1L is located in the dense tubular system, a remnant of the progenitor megakaryocyte ER (26, 27). The dense tubular system contains proteins known to be involved in protein folding, such as protein disulfide isomerase (28), and at least 1 of these has been shown to move to the platelet surface upon activation (29). SEL1L may migrate to the cell membrane upon activation in a similar manner.

Three additional model systems were used to further elucidate the role of SEL1L in platelets. SEL1L was investigated in human megakaryocyte cell culture. It was found to be expressed in different isoforms that increase in expression during megakaryopoiesis, regardless of the source of the hematopoietic progenitors. SEL1L may exist in different isoforms due to alternative mRNA splicing, proteolytic processing, and/or posttranslational modifications of the protein. For detection, we used an antibody capable of distinguishing these isoforms, focusing on 2 of the best-documented ones: SEL1L-p100kDa, corresponding to the full-length glycosylated form that acts as a central adapter in the ER-associated degradation (ERAD) machinery; and SEL1L-p38kDa, its shorter truncated isoform (30, 31). Both showed a significant increase in expression during megakaryopoiesis, with only SEL1L-p100kDa appearing to be passed on to mature platelets. Mouse megakaryocyte RNA-seq also revealed that *SEL1L* is expressed throughout megakaryocyte development and is passed on at low levels to mature platelets. The distribution of proteins from megakaryocytes to platelets is tightly regulated. Some of the key regulators of megakaryopoiesis and proplatelet formation, including ATXN2, P2Y13, and UDP-galactose-4-epimerase, have been shown to be primarily expressed in megakaryocyte progenitors rather than mature platelets (32–34). As such, while 2 SEL1L isoforms are expressed in megakaryocytes, the transfer of the larger isoform to platelets suggests it may play a functional role in mature platelets.

SEL1L was investigated in mouse and zebrafish models, demonstrating that it is necessary for proper platelet/thrombocyte adhesion to an injured vessel. Since the *SEL1L* KO is embryonic lethal in mice (35, 36), we took advantage of a conditional mutant. The mutant mouse tail bleeds also showed no significant difference in a bleeding time test, suggesting that SEL1L deficiency is not severe enough to be observed in this global assay. This agrees with the majority of the horse data (except the index case) and overall suggests this deficiency would result in a mild bleeding disorder. When we evaluated platelet function more specifically, we observed differences in the KO mice. There was a significant change in the number of platelets that adhered to immobilized collagen. There was some variability in the results, likely due to cre recombination efficiency (37) as well as normal biological variation in this assay (38, 39). Additionally, TEG revealed an association between SEL1L deficiency and a decrease in rate of clot development and strength. In contrast to the global mouse KO (35), we found that zebrafish with complete loss of SEL1L survive into adulthood. Previous work in zebrafish using morpholinos found that *sel1l* knockdown led to cardiac and brain edema, decreased blood circulation, and blood stasis in the tail (40), which suggested that SEL1L may play a role in circulation. Our mutants displayed no such phenotypes, suggesting the possibility that these morpholino phenotypes could be the result of off-target effects or genetic compensation allowing rescue in the KO (41).

To determine whether there was evidence for a role of SEL1L in human bleeding symptoms, we analyzed publicly available GWAS data. Two distinct substantial datasets revealed that variants in and proximal to the *SEL1L* locus are associated with several common bleeding phenotypes, including epistaxis and oral hemorrhage, consistent with the phenotype in the AET index case. These fall into the category of mucocutaneous bleeding, which is characteristic of platelet defects. These data support the hypothesis that SEL1L is required for normal platelet function and may contribute to human bleeding disorders. Given our data across models, it appears to be a mild bleeding disorder but may lead to severe hemorrhage when uncontrolled, as in the index AET case. *SEL1L* mutations would also likely modify other platelet or coagulation disorders.

We found that SEL1L plays a role in platelet activation and adhesion. In other cell types, it has been shown that the primary function of SEL1L is in ERAD (35, 36). SEL1L forms a complex with HRD1 (42) and is necessary for ER homeostasis and proper protein degradation and secretion (35, 36). A parallel and cooperative pathway to ERAD is the unfolded protein response (UPR) (43–45). UPR activation has been identified in mammalian platelets (46); thus, SEL1L could play a similar role in protein degradation in platelets. Recent studies have identified mechanisms of SEL1L modulating cell-signaling pathways by regulating the ubiquitination of a transcription factor and controlling the degradation of a specific receptor (47, 48). In a similarly indirect mechanism, SEL1L or HRD1 liver-specific deficiency in mice has been shown to cause aggregation of fibrinogen in inclusion bodies due to the hepatocytes’ inability to process misfolded fibrinogen through the ERAD pathway (49). Interestingly, in the SEL1L-KO model, circulating levels of fibrinogen were slightly decreased, and coagulation function (prothrombin time) was unchanged (49). In comparison, SEL1L deficiency in horses had no effect on circulating fibrinogen levels or basic coagulation function metrics (prothrombin time and activated partial thromboplastin time). Despite this small difference, SEL1L may have a similar mechanism in AET, where it regulates a protein involved in platelet function, likely in the thrombin signaling pathway since that is where differences were previously identified in AET-affected horses (2, 4).

In addition to SEL1L’s established role in ERAD, 2 other truncated isoforms of SEL1L have been shown to not solely localize to the ER in human lymphocytes (20). The largest isoform was predicted to be approximately 89 kDa, and the truncated ones were 34 and 35 kDa. Similarly, we identified isoforms of SEL1L in megakaryocytes of 100 and 38 kDa. The major isoform of 100 kDa is transferred from megakaryocytes to platelets at low levels and was confirmed to be present in the platelets/thrombocytes of all species in this study. Further study is necessary to determine if this isoform functions outside of the traditional ERAD pathway. It remains unknown whether a mutated SEL1L acts as a cell-autonomous or nonautonomous trait and how it affects other proteins or trafficking.

In summary, these results suggest that *SEL1L* (c.1810A>G p.Ile604Val) is responsible for the etiology of AET and a role for SEL1L in platelet function across 4 species. Although the similar assays employed had some differences due to species-specific limitations, the results corroborated each other (Supplemental Table 4). SEL1L appears to be necessary for platelets or thrombocytes to properly adhere to sites of injury through interaction with collagen, and loss of function impacts hemostasis. This identification of the putatively causative variant of AET will allow for affected TBs and carriers to be identified and inform breeding decisions. Additionally, this work has identified SEL1L, and possibly the ERAD pathway, as important players in platelet function, with GWAS data supporting that there may be implications for patients with unknown bleeding disorders.

## Methods

### Sex as a biological variable.

Our experiments used both male and female animals as there was no difference in phenotype between sexes.

### Horses and whole-genome sequencing.

Seventeen TBs (5 affected and 12 unaffected) were phenotyped in a previously published study (3). Briefly, platelets were activated with thrombin, and the amount of fibrinogen bound was quantified via flow cytometry and compared with the average of 3 unaffected horses (2). A strict cutoff of an average of 35% of fibrinogen bound by platelets or less compared with control horses was used to definitively diagnose affected horses. Whole-genome sequencing was performed on the entire group using an Illumina HiSeq 2500, at a targeted 50× coverage with paired-end 150 bp reads. Raw reads were trimmed with Trimmomatic (50), mapped to the EquCab3.0 reference genome (51) using Burrows-Wheeler Aligner (BWA-MEM) (52), and sorted by coordinate with samtools (53). PCR duplicates were removed with Picard tools (http://broadinstitute.github.io/picard/). Integrative Genome Viewer (54), was used to genotype all horses for the 2 variants shown to cause Glanzmann thrombasthenia (5–7). Freebayes (55) was used to call single nucleotide variants and small insertions/deletions. Delly was used to identify larger structural variants (56). The functional effects of the variants were predicted using SnpEff (57), and the variants were filtered by segregation using Fischer’s exact test with SnpSift (58). Associated variants were identified and filtered by predicted effect. PlateletWeb (http://plateletweb.bioapps.biozentrum.uni-wuerzburg.de/plateletweb.php) was used to identify the variants that are in and near genes known to be expressed in human platelets. KaryoploteR (59) was used to identify haploblocks among the variants that were significantly different (*P* < 0.0001).

### Candidate gene approach.

Candidate genes (*AKT1*, *AKT2*, *F5*, *SHIP1*, *PIK3C2B*, *F2, F2R*, *PIK3C2A*, *PIK3C2G*, *PIK3CA*, *PIK3CB*, *PIK3CG*, *PIK3R1*, *PIK3R4*, *PIK3R5*, and *PIK3R6*) were identified from previous biochemical work (4) on AET-affected platelets (Supplemental Figure 1). One kilobase of sequence up and downstream was included to account for the 5′ and 3′ untranslated regions that could harbor regulatory variants. The functional effects of the variants were predicted using SnpEff (57), and variants were filtered by segregation using Fisher’s exact test with SnpSift (58).

### Equine blood processing.

Blood samples were obtained from TBs at the University of California, Davis, Center for Equine Health (UCD CEH). Blood was drawn into acid citrate dextrose tubes, and platelets were separated from whole blood by density centrifugation and washed to yield washed platelets. To remove leukocytes, the washed platelets were treated with prostaglandin E1 and depleted of leukocytes using an anti-equine F6 antibody provided by Jeff Stott (University of California, Davis).

### RNA isolation, RT-PCR, and RT-qPCR.

RNA was extracted from leukocyte-depleted platelets using phenol-chloroform phase separation, as previously described (60), and treated with Turbo DNase (Thermo Fisher Scientific). Superscript III (Thermo Fisher Scientific) was then used to reverse transcribe 500 ng of RNA. Primers that yield products spanning 2 exons were designed for *SEL1L*, *AL355838.1*, and *VIPAS39* (Supplemental Table 1), and end-point PCR was performed on a sample from a WT horse.

cDNA from leukocyte-depleted platelets (*SEL1L A>G*: *n* = 12 WT, 6 heterozygous, and 2 homozygous; *VIPAS39del*: *n* = 16 WT, 3 heterozygous, and 1 homozygous) was run in triplicate using SYBR green on an AriaMx Real-Time PCR System (Agilent). The *ACTB* gene was used for normalization (Supplemental Table 1), and ΔΔCt values were calculated.

### Genotyping additional horses.

114 phenotyped (3) TBs were genotyped for *SEL1L* A>G using PCR-RFLP with the restriction enzyme HpyCH4III or for the *VIPAS39* deletion by visualization on an agarose gel (Supplemental Table 1).

### Flow cytometry of equine platelets.

Washed equine platelets (*SEL1L A>G*: *n* = 7 WT, 5 heterozygous, and 2 homozygous alternate) were fixed in 1% paraformaldehyde, permeabilized in 0.1% NP-40, and labeled with a monoclonal SEL1L antibody conjugated to AlexaFluor488 (Santa Cruz Biotechnology; sc-377350) and a monoclonal P selectin antibody conjugated to APC (Invitrogen; 17-0626-82). Samples were analyzed on a Beckman Coulter FC500 flow cytometer, and data were analyzed with FlowJo.

### TBTs.

TBTs were measured on 13 horses (10 WT and 3 homozygous alternate) at the UCD CEH as previously described (61). Briefly, a template system (MedexSupply) was used on a clipped area just distal to the accessory carpal bone on the caudomedial and caudolateral aspects of the forelimbs. A sphygmomanometer cuff was placed proximal to the carpus and inflated to 40 mmHg for 60 seconds to achieve cutaneous venostasis. Blood flow from the incision was collected on grade 40 absorbent filter paper at 30-second intervals until no capillary flow was absorbed. Bleeding times were performed at a standardized time each morning on both forelimbs over 3 consecutive days and mean times determined. Blood samples for complete blood count and basic coagulation panel (prothrombin time, activated partial thromboplastin time, and fibrinogen) were obtained prior to the study to ensure no influences by changes in platelet count or clotting defects.

### Immunofluorescence.

Resting and thrombin-activated (bovine α-thrombin; Haematologic Technologies), washed TB platelets (10 × 10^6^ platelets [plt]/mL) were incubated on poly-l-lysine coverslips (37°C/1 h). The platelets were then fixed with 1% paraformaldehyde, permeabilized with 0.1% NP-40, and blocked with 10% BSA. Platelets were incubated with anti–P selectin (BD Pharmaceuticals; 553742, diluted 1:10 in 5% goat serum), followed by a secondary antibody conjugated to an AF488 fluorophore (Thermo Fisher Scientific; A11006, diluted 1:20 in 5% goat serum). Both incubations were performed for 2 hours, rocking, at room temperature (RT). Next, fixed platelets were incubated with a cocktail of anti-SEL1L and anti-CD42b (Abcam; ab78298 and ab9505-500, diluted 1:200 and 1:10, respectively, in 5% goat serum), rocking overnight at 4°C. Lastly, the platelets were incubated with a cocktail of secondary antibodies conjugated to AF555 and AF405 (Thermo Fisher Scientific; A32732 and A31553, diluted 1:300 and 1:10, respectively, in 5% goat serum) for 2 hours, rocking, at RT. The stained platelets were imaged on a Leica TCS SP8 STED 3X (Leica Microsystems).

### Collagen spreading assay.

Washed platelets (9 × 10^9^ plt/L) were allowed to adhere to type I collagen–coated coverslips (Electron Microscopy Sciences; 37°C/2 h), washed in buffer, and fixed in 1% paraformaldehyde (RT/1 h). The mean platelet volume of the platelet-rich plasma (PRP; *n* = 11 WT, 5 heterozygous, and 2 homozygous alternate) was determined. The platelets were washed and allowed to adhere to type I collagen–coated coverslips (9 × 10^9^ plt/L; Electron Microscopy Sciences; 37°C/2 h). These were subsequently washed in buffer, fixed in 1% paraformaldehyde (RT/1 h), and mounted. The platelets were imaged using an Olympus BX62 microscope. Platelet area was determined using ImageJ 1.51K (NIH) by an observer blinded to genotype.

### Human megakaryocyte differentiation.

Umbilical cord blood units were collected in bags containing acid-citrate-dextrose (ACD). Adult human samples were obtained from healthy donors. Megakaryocytes were differentiated from human CD34^+^ cells using previously described methods (16,17). Briefly, CD34^+^ cells were separated by an immunomagnetic sorting technique (17). The purity of the sorted population was routinely assessed by flow cytometry to be 90 ± 4% of the total cells recovered. CD34^+^ cells were cultured for 13 days in Stem Span media (STEMCELL Technologies) supplemented with 10 ng/mL TPO and interleukin-11 (Peprotech); 1% penicillin-streptomycin and 1% L-glutamine (Euroclone). The culture medium was renewed every three days.

### Isolation of human platelets from peripheral blood.

For SEL1L protein expression analysis, human whole blood from healthy subjects was collected using ACD and processed as previously described (62). Briefly, platelets were isolated by centrifugation at 200g for 10 minutes to obtain PRP. PRP was then centrifuged at 1500g for 15 minutes in presence of 0.2 U/mL apyrase grade I and 1 μm PGE1 (Sigma-Aldrich). Platelets were then gently resuspended in Tyrode’s buffer (134 mM NaCl; 0.34 mM Na_2_HPO_4_; 2.9 mM KCl; 12 mM NaHCO_3_; 20 mM HEPES; 5 mM glucose; pH 6.5). Washed platelets were finally lysed with Hepes-glycerol lysis buffer (Hepes 50 mM, NaCl 150 mM, 10% glycerol, 1% Triton X-100, MgCl_2_ 1.5 mM, EGTA 1 mM, NaF 10 mM, PMSF 1 mM, Na_3_VO_4_ 1 mM, 1 μg/mL leupeptin, 1 μg/mL aprotinin). Before being lysed, purity of the platelet samples was assessed by flow cytometry, as previously described (63).

To compare *SEL1L-A* mRNA isoforms, whole blood was collected from healthy human donors via venipuncture into vacutainer tubes containing 3.2% sodium citrate (Greiner Bio-One). PRP was isolated by centrifuging the whole blood at 200*g* for 15 minutes. ACD (10×; 2.5% sodium citrate tribasic [C_6_H_9_Na_3_O_9_], 1.5% citric acid [C_6_H_8_O_7_], and 2% d-glucose) and apyrase (0.02 U/mL) were added to PRP to prevent platelet activation. PRP was then centrifuged at 2,000*g* for 10 minutes to pellet the platelets. The platelet-poor plasma (PPP) supernatant was aspirated and discarded. The remaining platelet pellet was frozen using liquid nitrogen and stored at –80°C.

### Immunoblotting.

Human platelet and megakaryocyte protein lysates were subjected to gel electrophoresis (SDS-PAGE) and transferred to a PVDF membrane (Bio-Rad). Membranes were probed with mAb anti-SEL1L (64) (1:200) and mAb anti–β-actin (Abcam; ab6276, 1:1,000). Immunoreactive bands were detected by horseradish peroxidase–labeled secondary antibodies (Bio-Rad; 1706516 and 1706515) using enhanced chemiluminescence reagent (Millipore). Prestained protein ladders (Bio-Rad) were used to estimate the molecular weights. Band intensities from individual Western blots were quantified by densitometric analysis using ImageJ software.

### Analysis of proplatelet formation.

Megakaryocyte and proplatelet yields were evaluated at the end of the cell culture as previously described (17). Briefly, 1 × 10^5^ cells were seeded in a 24-well plate and incubated at 37°C in a 5% CO_2_ fully humidified atmosphere. Proplatelets were identified as cells displaying long filamentous structures, ending with platelet-sized tips. Phase contrast images were obtained by an Olympus IX53 microscope.

For the immunofluorescence analysis, mature megakaryocytes were seeded onto 12 mm glass coverslips coated with 25 μg/mL human fibronectin (Corning) for 2 hours at RT and subsequently blocked with 1% albumin (Sigma-Aldrich) for 1 hour at RT. Megakaryocytes were harvested and plated onto coated coverslips in 24-well plates and allowed to adhere for 16 hours at 37°C in a 5% CO_2_ fully humidified atmosphere. Next, cells were fixed in 4% paraformaldehyde (Sigma-Aldrich), permeabilized with 0.5% Triton X-100 (Sigma-Aldrich), blocked with 5% BSA, and stained with anti-SEL1L (64) (1:200) and/or anti–β1-tubulin antibodies (Abcam; ab179511, 1:1,000) for 1 hour at RT and the Alexa Fluor secondary antibodies (Invitrogen; A-11034 and A-11032, 1:500) for 2 hours at RT. Nuclear counterstaining was performed using Hoechst 33258 (100 ng/mL; Sigma-Aldrich) for 3 minutes at RT. Specimens were mounted in ProLong Gold Antifade Reagent (Invitrogen). Negative controls were routinely performed by omitting the primary antibody. Immunofluorescence images were acquired by a Nikon Ti2 microscope. Megakaryocytes extending proplatelets were identified as cells extending tubulin-positive long filamentous structures ending with platelet-sized tips.

### Flow cytometry analysis of megakaryocytes.

For megakaryocyte differentiation analysis, cells were suspended in PBS buffer and stained with FITC anti-CD61 and PE anti-CD42b antibodies (Beckman Coulter; IM1758 and IM1417U) at RT in the dark for 30 minutes. All samples were acquired with a BD Biosciences flow cytometer. Offline data analysis was performed using Kaluza software.

### Analysis of mouse Sel1l expression.

Two previous RNA-seq experiments (18, 19, 65) were used to evaluate SEL1L expression across mouse megakaryocyte development and in human and mouse platelets.

### Mouse blood collection and platelet isolation.

Blood was drawn from the inferior vena cava of anesthetized male and female WT C57BL/6J mice (The Jackson Laboratory) 10–14 weeks of age. The blood was drawn into a syringe containing 3.8% sodium citrate at a 9:1 ratio of blood/citrate. PRP was isolated by serial centrifugation. Briefly, whole blood was centrifuged at 200*g* for 5 minutes. PRP supernatant was transferred to a new vial, and 500 μL of Tyrode’s buffer was added, followed by centrifugation at 200*g* for 5 minutes. This was repeated twice, and the resulting PRPs from 4 mice were pooled. The pools were treated with acid citrate dextrose (10×) and apyrase (0.02 U/mL). The PRP was then centrifuged at 2,000*g* for 10 minutes to pellet the platelets. The PPP supernatant was aspirated and discarded. The pellet was frozen using liquid nitrogen and stored at –80°C.

### Platelet/thrombocyte SEL1L-A expression across species.

RNA from washed platelets from WT horse, human, and mouse was isolated as described above and reverse transcribed into cDNA. 5 dpf *cd41-eGFP* transgenic zebrafish larvae (66) with fluorescent thrombocytes were dissociated in 0.25% trypsin-EDTA and proteinase K (0.2 mg/mL) and processed as previously described (67). GFP^+^ thrombocytes were isolated through FACS using a BD FACSDiscover S8 Cell Sorter, and RNA was isolated and reverse transcribed into cDNA as described above. RT-PCR was performed with primers based on previous work to amplify *SEL1L-A* (20, 68) (Supplemental Table 1). The resulting PCR product was Sanger sequenced and compared with the appropriate reference genome using BLAT (BLAST-like alignment tool) on the UCSC Genome Browser (68, 69).

### Murine SEL1L conditional knockdown.

*Mx1-cre^+^ Sel1L^fl/fl^* and *Mx1-cre*^−^ *Sel1L^fl/fl^* mice (36, 37) were treated at 10 weeks with poly-inosine-poly-cytosine (pIpC) as previously described (37). Two weeks after the last pIpC treatment, cre^+^ and cre^−^ paired littermates were used for all studies.

### Mouse tail bleeding assay.

pIpC-treated *Mx1-cre^+^ Sel1L^fl/fl^* and *Mx1-cre*^−^ *Sel1L^fl/fl^* mice were anesthetized with ketamine/xylazine via i.p. injection and placed on a heating pad. A scalpel was used to cut 5 mm off the end of each tail, and the remaining tail was immediately placed in a tube of saline heated to 3°C and bleeding time recorded, which was determined as previously described (70, 71). Briefly, the time to cessation of bleeding was recorded, and, if rebleeding occurred after less than 1 minute, the additional time was added to the total. Experiments were terminated at 10 minutes after tail resection regardless of bleeding cessation due to ethical considerations. Following either cessation of bleeding or termination of the experiment, the tails were cauterized using a high-temperature cautery (Bovie Medical), and the mice were dosed with carprofen (5 mg/kg) for analgesia. Mice were kept under warm lights until they recovered from the anesthetic, and the cautery wound was assessed to ensure no additional bleeding. Mice were given free access to food and water, and their tails were monitored for 1 week during recovery.

### Platelet perfusion over collagen.

Whole blood was collected from pIpC-treated *Mx1-cre^+^ Sel1L^fl/fl^* and *Mx1-cre*^−^ *Sel1L^fl/fl^* littermates through the retro-orbital space into sodium citrate. The citrated blood was incubated with DiOC_6_ (Thermo Fisher Scientific) for 5 minutes at 3°C. Platelet adhesion was measured as previously described (38, 72). Briefly, flow chamber μ-slides (1 × 17 mm; Ibidi) were coated with 50 μg/mL equine collagen (Chronolog) overnight at 4°C and rinsed with 37°C 1× PBS. The blood was perfused over the collagen-coated chamber at 1,800 s^−1^, a typical arterial shear rate, by an automated syringe pump. Adhesion of the fluorescently labeled platelets was recorded using a Zeiss Axiovert 200M inverted fluorescent microscope at 40×. The videos were analyzed using 3i Slidebook. The mean fluorescence was measured at the beginning and end of perfusion. The difference was determined and normalized to the cre^−^ mouse of each pair.

### TEG.

pIpC-treated *Mx1-cre^+^ Sel1L^fl/fl^* and *Mx1-cre*^−^ *Sel1L^fl/fl^* siblings were anesthetized using inhaled isoflurane, and blood was drawn via the inferior vena cava. The blood was placed in 3.8% sodium citrate at a 9:1 blood/sodium citrate ratio. The blood was recalcified with 0.2 M CaCl_2_ prior to performing TEG using a Haemoscope TEG 5000 Thromboelastograph Hemostasis Analyzer (Haemonetics Corp.). Coagulation parameters including R time (time to initiation of fibrin clot formation), α-angle (rate of clot formation), K time (the time until the clot reaches a strength of 20 mm), maximum amplitude (maximum clot strength), and maximum rate of thrombin generation were analyzed and compared.

### Development of sel1l-KO zebrafish.

A *sel1l-*KO zebrafish line was generated on a WT background of a hybrid of AB and TL strains (AB × TL; Zebrafish International Resource Center). Synthego’s CRISPR Design Tool was used to identify CRISPR/Cas9 sgRNA target sites in exons 3 (GAAGGUGCCAAUAUUGUGAC) and 20 (GGAUCUCAAUGUAAGUUGAG). The sgRNA was complexed with Cas9 (Synthego), injected into 1-cell embryos, and raised to adulthood. The fish were genotyped and crossed to *cd41-eGFP* transgenic fish (66) to establish a line with fluorescently labeled thrombocytes.

### Laser-mediated endothelial injury in larval zebrafish.

At 5 dpf, laser-mediated arterial endothelial injury was performed as previously described (73, 74). Briefly, the larvae were anesthetized with tricaine and mounted with 0.8% low-melting-temperature agarose on a glass coverslip. Endothelial injury was performed with a pulsed nitrogen dye laser system (Andor Technology) and analyzed on an inverted microscope (Olympus IX73) using a ×20 objective. The endothelium of the dorsal aorta at the fifth somite posterior to the anal pore was targeted with the laser. *cd41-eGFP*^+^ thrombocytes were visible under fluorescence, and the time to first thrombocyte attachment was measured (up to 2 minutes). The total number of thrombocytes that attached and time to full occlusion (up to 2 minutes) were also measured. The larvae were genotyped after the data were collected.

### Thrombocyte counting.

Relative thrombocyte count was measured as previously described (75). Briefly, anesthetized 5 dpf larvae were immobilized in 0.8% low-melt agarose in a glass capillary. The capillary was submerged in water and placed under a Leica MZ16FA stereomicroscope with GFP fluorescence. A 1-minute video was recorded, and VirtualDub (https://www.virtualdub.org/) was used to transform the videos into grayscale. ImageJ was used to analyze the relative thrombocyte count in each video.

### Investigating SEL1L in human GWAS.

The PheWeb platform (21) was used to identify complex bleeding phenotypes associated with loci in or proximal to *SEL1L*. The databases that were investigated were from The Michigan Genomics Initiative biobank, which included data from 51,583 individuals (22), and the Neale lab (https://pheweb.org/UKB-Neale/) using data from the UK Biobank, which contained genotypes from 337,199 individuals.

### Statistics.

Data analysis was performed using GraphPad Prism software. Parametric data were analyzed with a 1-way ANOVA or a 2-tailed Student’s *t* test. Nonparametric data were analyzed with a Mann-Whitney *U* test, a Kruskal-Wallis test with multiple comparisons, and a FDR correction or Wilcoxon’s matched-pairs signed-rank test. The GWAS included logistical regression analysis to determine association. *P* < 0.05 was considered a statistically significant difference.

### Study approval.

All horse experiments were conducted in accordance with guidelines approved by the University of California, Davis, IACUC. For all human megakaryocyte experiments, written informed consent was obtained at enrollment in accordance with the ethical committee of the IRCCS Policlinico San Matteo Foundation of Pavia and the principles of the Declaration of Helsinki. Human blood for platelet isolation was obtained with written informed consent and the approval of the University of Michigan IRB. All mouse and zebrafish experiments were done in accordance with guidelines approved by the University of Michigan IACUC.

### Data availability.

All sequencing reads for this study have been submitted to NCBI Sequence Read Archive under PRJNA553581. Full VCF files for each candidate gene were submitted to the European Variation Archive under PRJEB33757. The variant *SEL1L* (c.1810A>G p.Ile604Val) has been added to the Online Mendelian Inheritance in Animals database with the identifier omia.variant:1819. Values for all data points in graphs are reported in the Supporting Data Values file.

## Author contributions

ARD designed research studies, conducted experiments, acquired and analyzed data, and wrote the manuscript. FC conducted experiments and acquired and analyzed data. JWN and LS designed research studies, conducted experiments, and acquired and analyzed data. CADB conducted experiments, acquired and analyzed data, and edited the manuscript. RA designed research studies, conducted experiments, and acquired data. EMK conducted experiments, acquired data, and provided reagents. QL and MH designed research studies and provided reagents. IB designed research studies, analyzed data, and provided reagents. AB designed research studies, analyzed data, provided reagents, and edited the manuscript. JAS and CJF designed research studies, provided reagents, and edited the manuscript. FT designed research studies, conducted experiments, acquired and analyzed data, provided reagents, and edited the manuscript.

## Funding support

This work is the result of NIH funding, in whole or in part, and is subject to the NIH Public Access Policy. Through acceptance of this federal funding, the NIH has been given a right to make the work publicly available in PubMed Central.

- NIH grant T32 HL007622 (to JAS).
- NIH grant R35 HL150784 (to JAS).
- NIH grant R01 ES 032255 (to JAS).
- NIH grant 1K01OD015134 (to CJF).
- Ann T. Bowling Fellowship (to ARD).
- Center for Equine Health (to ARD).
- Louis R. Rowan Fellowship (to ARD).
- Boxer Training Program in Molecular and Translational Hematology (to ARD).
- Henry and Mala Dorfman Family Professor of Pediatric Hematology/Oncology (to JAS).
- European Hematology Association (RG-202012-00212; to CADB).
- European Innovation Council Transition Project SilkPlatelet (project 101058349; to IB and AB).

## Supplementary Material

Supplemental data

Unedited blot and gel images

Supporting data values

## Figures and Tables

**Table 1:**
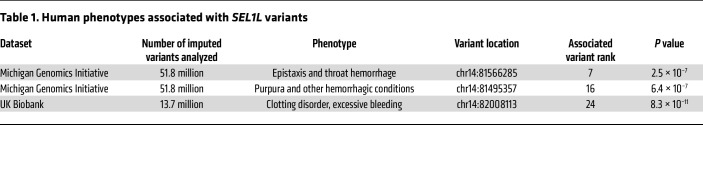
Human phenotypes associated with *SEL1L* variants

## Version 1 02/16/2026

Electronic publication
