# Supplementary material for: ER-associated degradation pathway protein SEL1L plays an evolutionarily conserved role in platelet adhesion
Source: J Clin Invest. 2026 Feb 16;136(4):e191433. doi: 10.1172/JCI191433 (PMC12904699; doi:10.1172/JCI191433)
Supplement: Supplemental data [file jci-136-191433-s164.pdf]

Supplemental Figures

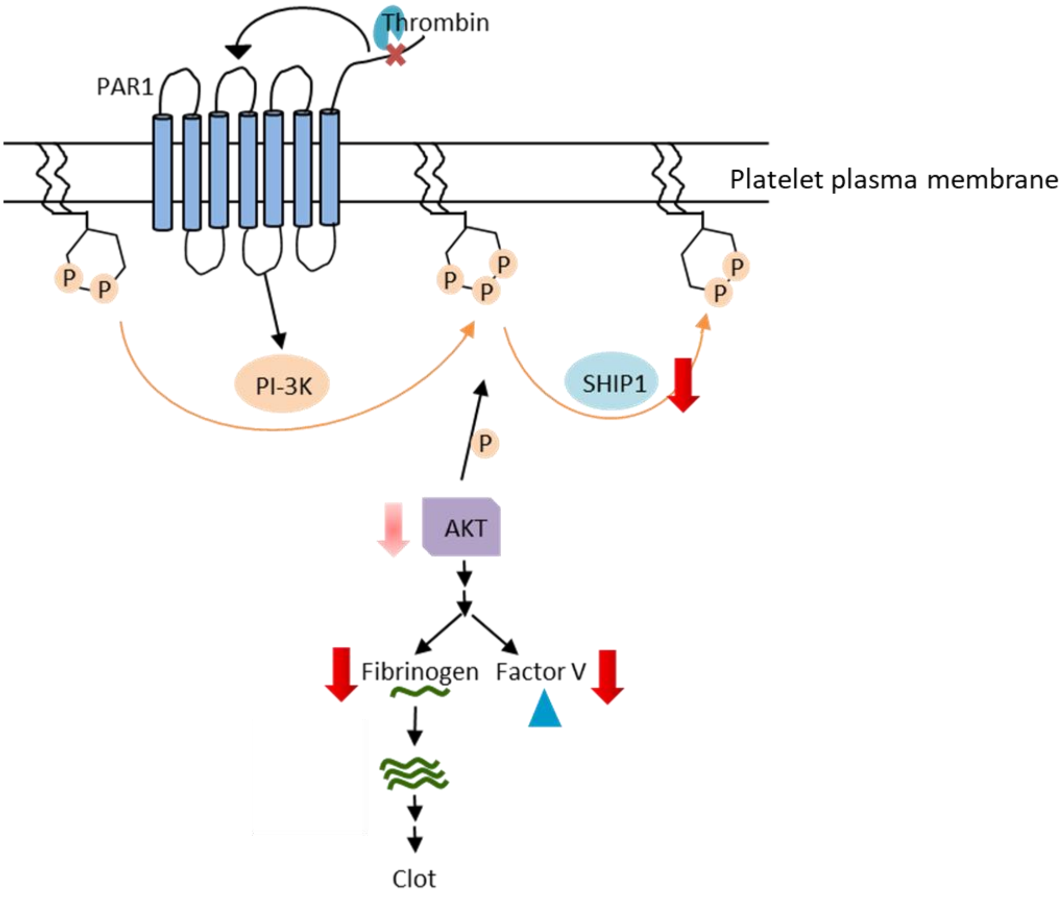

Supplemental Figure 1: Thrombin signaling in platelets. Changes in protein expression in AET-affected horses are indicated by red arrows. SHIP1 levels are decreased in the platelet membrane, and an increase in association of PIK3C2B with the membrane were seen in AET-affected horses. There is abnormal phosphorylation of AKT after activation as well as decreased levels of fibrinogen bound by activated platelets. Additionally, the amount of FV released is decreased in affected horses.

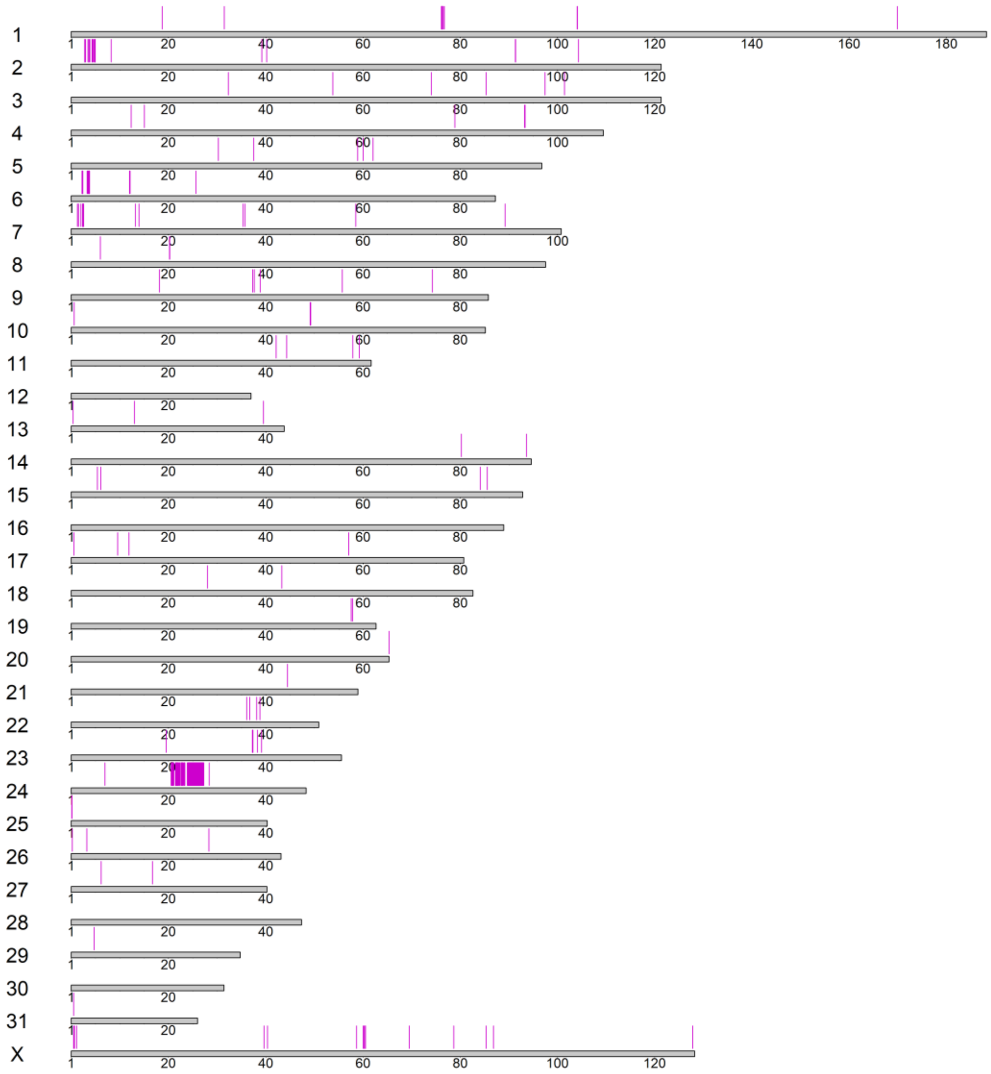

Supplemental Figure 2: Visualization of AET-associated variants across the equine genome. The representation of 3,769 Freebayes variants across the horse genome where each chromosome is represented by a grey bar and the magenta lines indicate variant positions. A large ~6Mb haploblock was identified on chromosome 24 (21Mb – 27Mb).

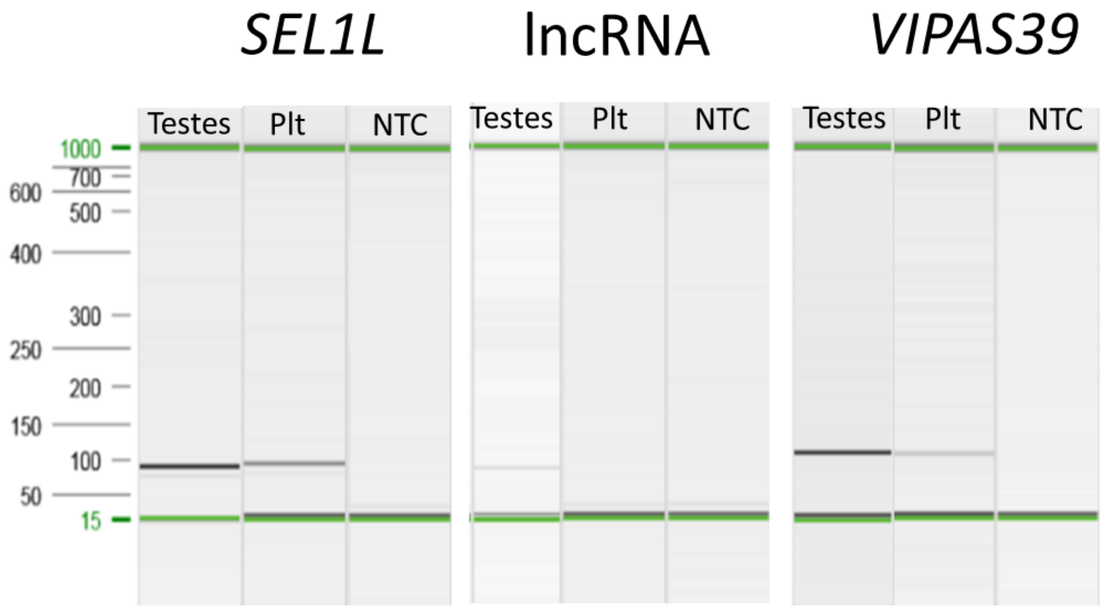

Supplemental Figure 3: *SEL1L* mRNA is expressed in horse platelets. Reverse transcription PCR showing transcript presence in testes (positive control) and platelets (plt). NTC represents no template control. *SEL1L* and *VIPAS39* transcripts are shown to be present in platelets while the lncRNA transcript is not.

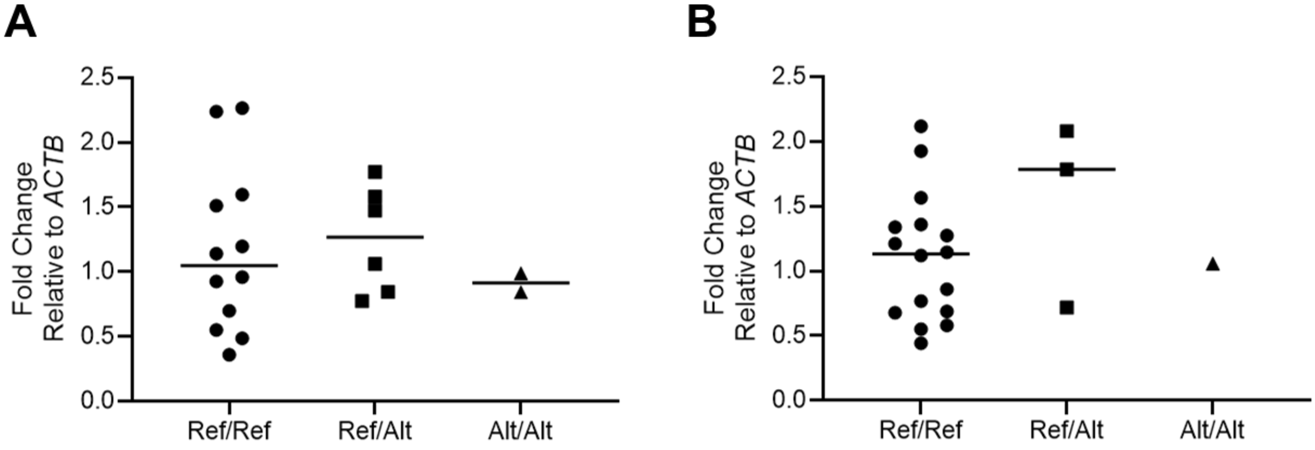

Supplemental Figure 4: The Alt *SEL1L* mRNA is expressed at levels comparable to the Ref allele. Quantitative reverse transcription PCR from platelet mRNA for (**A**) *SEL1L* (n=12 Ref/Ref, 6 Ref/Alt, 2 Alt/Alt) (**B**) *VIPAS39* (n= 16 Ref/Ref, 3 Ref/Alt, 1 Alt/Alt) by genotype. Fold change relative to *ACTB* is shown with the median indicated by a horizontal line. There were no significant differences between genotypes as determined by a Kruskal-Wallis test.

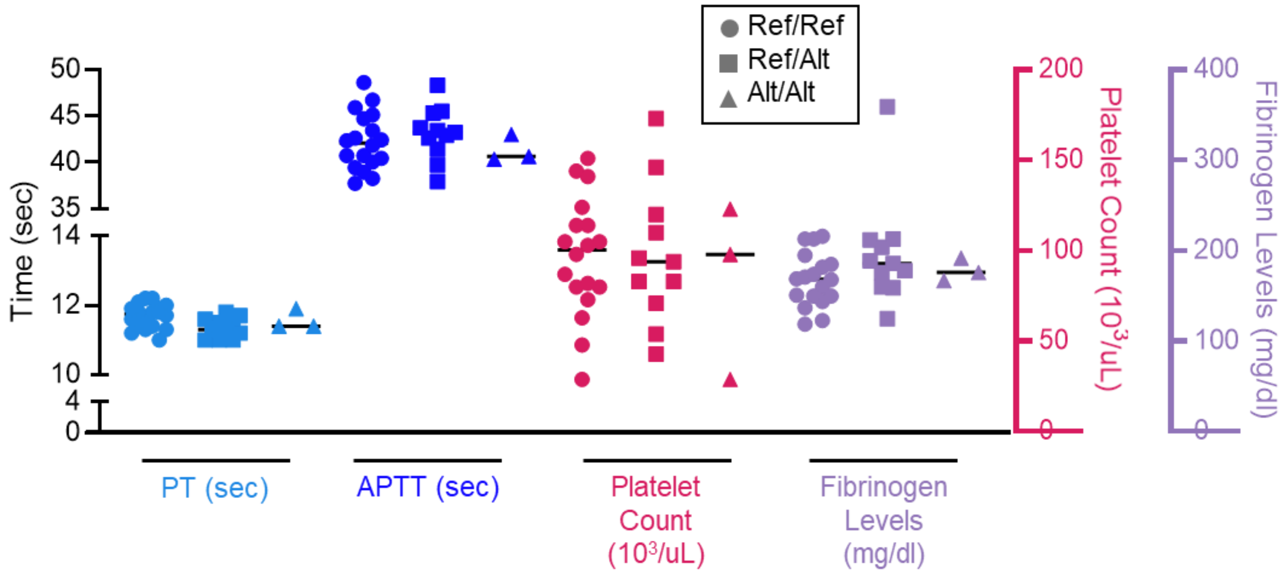

Supplemental Figure 5: Clinical coagulation studies are normal in AET horses. Results of prothrombin (PT), activated partial thromboplastin time (APTT), platelet count, and fibrinogen quantification in a larger horse population indicating that there are no other severe clotting disorders that may contribute to the phenotype.

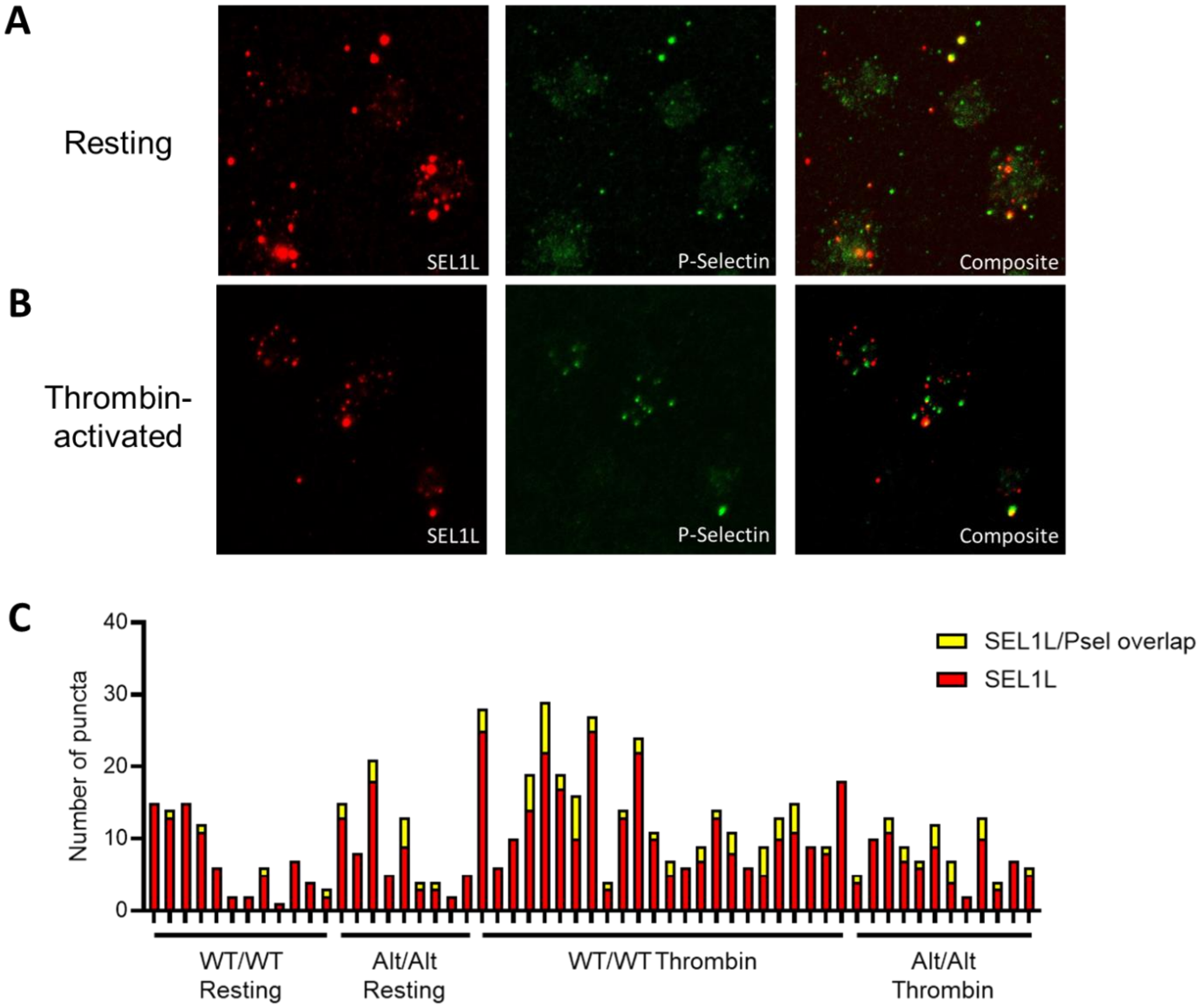

Supplemental Figure 6: SEL1L localization in equine platelets. SEL1L (red) localization in relation to P-selectin (Psel, green) in (**A**) resting permeabilized platelets and (**B**) thrombin activated platelet surface of an Alt/Alt horse. SEL1L did not localize to the surface in resting permeabilized platelets but did localize to the surface in thrombin-activated platelets. N=2 horses. (**C**) Quantification of the number of yellow (SEL1L/Psel overlap) and red (SEL1L) puncta by an observer blinded to condition with ImageJ demonstrates minimal overlap and no significant difference between genotypes or activation status. P=0.09 as determined by Kruskal-Wallis test.

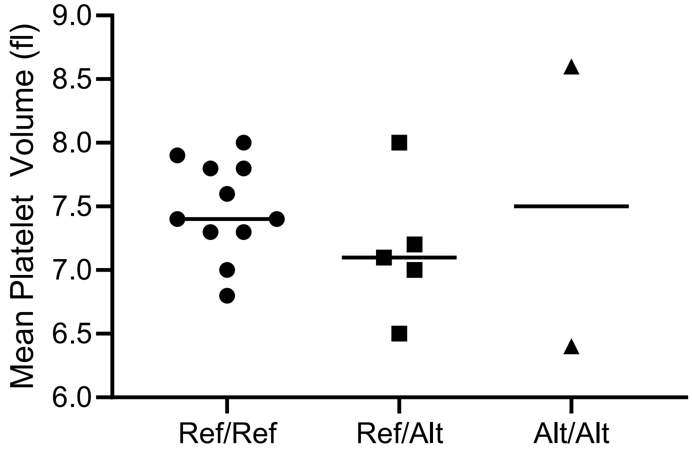

Supplemental Figure 7: No difference in mean platelet volume of *SEL1L* deficient equine platelets. Mean platelet volumes from platelet rich plasma for each individual horse separated by genotype for *SEL1L* c.1810A>G p.Ile604Val. No significant difference was detected among the genotypes. N=11 Ref/Ref, 5 Ref/Alt, 2 Alt/Alt.

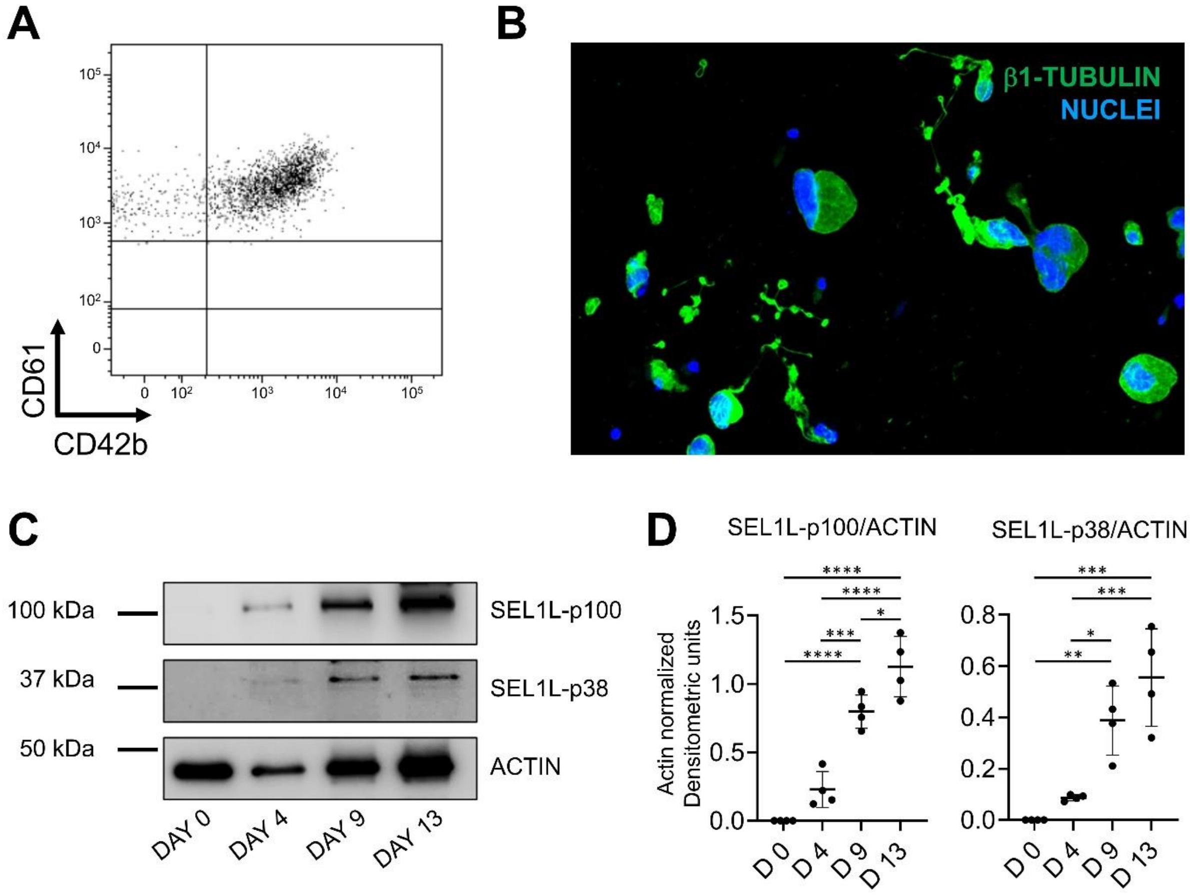

Supplemental Figure 8: SEL1L is expressed in human adult blood megakaryocytes and platelets. (**A**) Representative flow cytometry analysis of human mature megakaryocytes differentiated from adult stem and progenitor cells for 2 weeks in the presence of thrombopoietin. (**B**) Representative immunofluorescence microscopy of megakaryocytes extending proplatelets (scale bar = 15 μM). (**C**) Representative western blot analysis of SEL1L during adult megakaryopoiesis. (**D**) Densitometric analyses of SEL1L isoforms (n=4, results are presented as mean ± SD). * *P*<0.05; ** *P*<0.001 by one-way ANOVA with post-hoc pairwise comparisons.

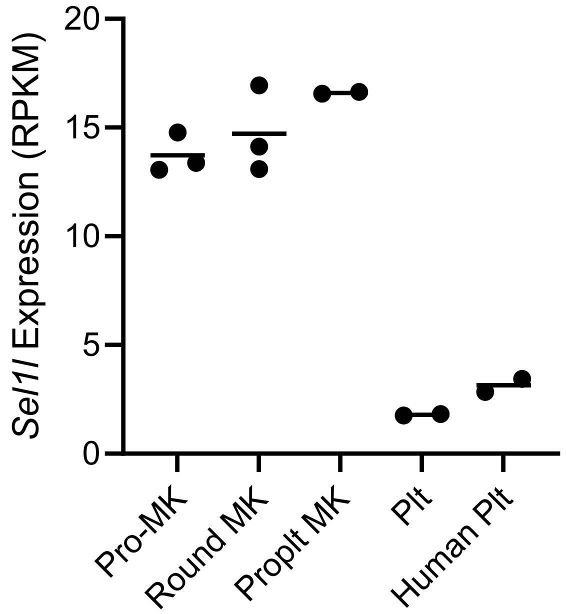

Supplemental Figure 9: RNA-seq of mouse megakaryocytes and platelets reveals *Sel1l* mRNA expression. *Sel1l* mRNA expression in reads per kilobase million (RPKM) from RNA-seq of megakaryocytes (MK) across development into platelets (plt), shows expression at each stage. *SEL1L* expression in human platelets is present at similarly low levels to mouse platelets. The line indicates the mean.

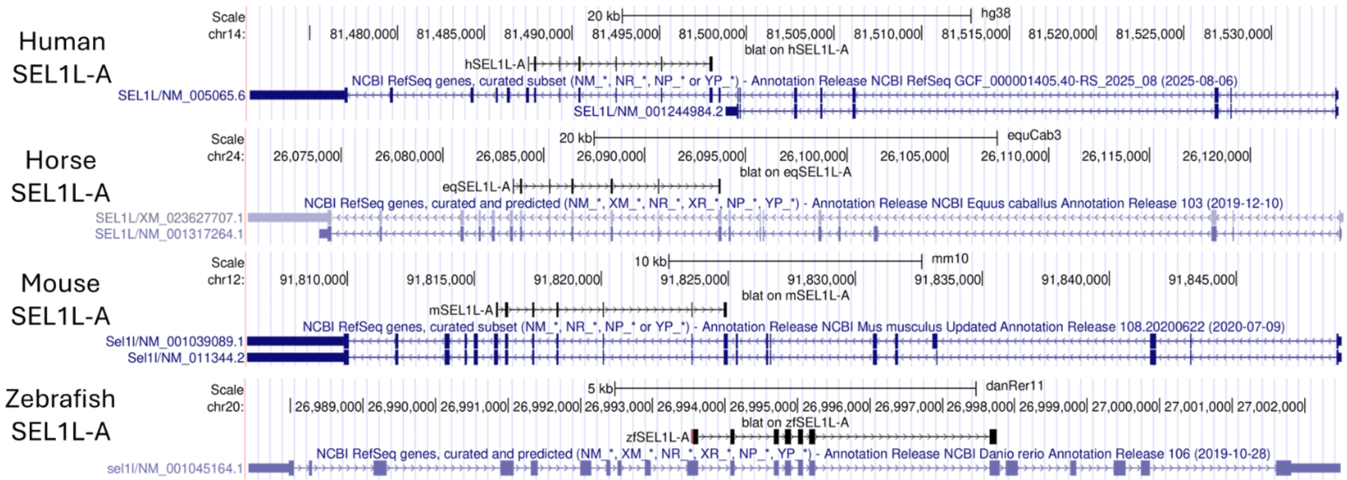

Supplemental Figure 10: RT-PCR of *SEL1L-A* in human, horse, mouse and zebrafish platelets/thrombocytes. RT-PCR results for *SEL1L-A* aligned to the appropriate reference genome using the UCSC genome browser. The blue tracks represent annotated *SEL1L* transcripts. The black tracks demonstrate that *SEL1L-A* is expressed in each species.

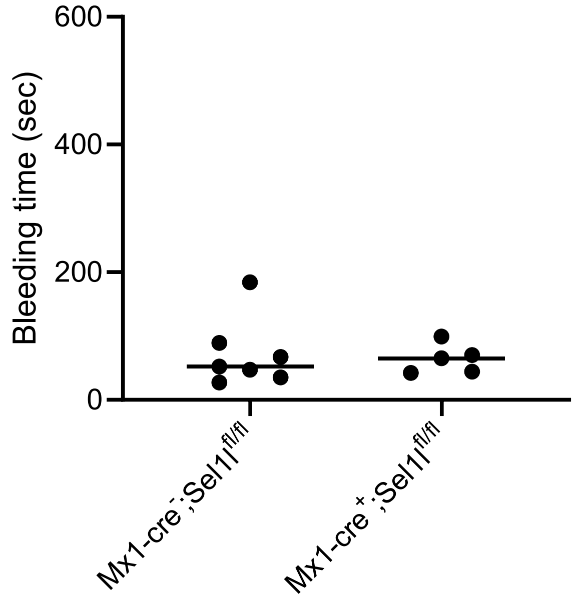

Supplemental Figure 11: Tail bleeding time is unchanged in *Sel1l* mutant mice. Tail bleed times in seconds for control *Mx1-cre*$^{-}$*;Sel1l*$^{fl/fl}$ (N=7) and knockdown *Mx1-cre*$^{+}$*;Sel1l*$^{fl/fl}$ (N=5) mice. Mann-Whitney *U* testing showed no significant difference between the groups.

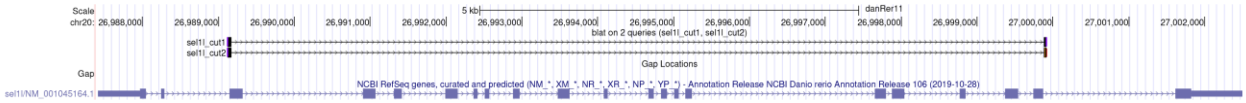

Supplemental Figure 12: Depiction of the CRISPR-Cas9 mediated deletion in zebrafish *sel1l*. The annotation of the *sel1l* gene is shown in the blue track. The deletion, as depicted by the black bar, spans from exon 3 to exon 20.

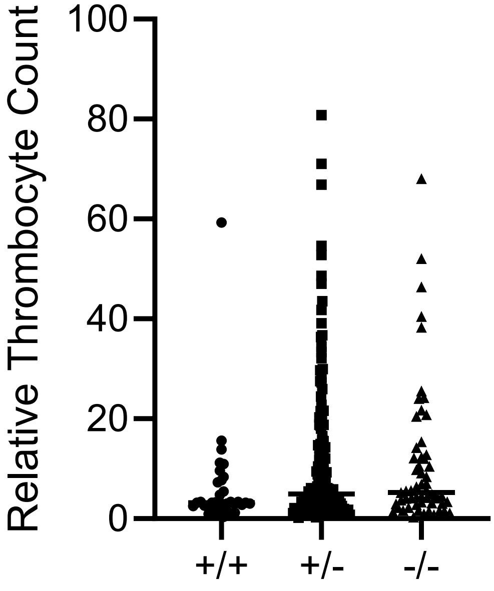

Supplemental Figure 13: Thrombocyte counts are unchanged in *sel1l* knockout zebrafish. Relative thrombocyte counts of wild-type (+/+), heterozygous (+/-), and homozygous (-/-) mutant offspring at 5 dpf from a heterozygous incross of the zebrafish *sel1l* knockout shows no difference in average counts between genotypes. N=36 +/+, 154 +/-, 58 -/-. Not significant by ANOVA testing.

**Supplemental Table 1: Primer Sequences**			
**Primer Target**	**Coordinates**	**Forward**	**Reverse**
SEL1L transcript	chr24:26,074,490-26,076,942	CCTAGCACTCTGCAAATTAGGC	CAAGTTGGGTGAAGACATCTCG
VIPAR transcript	chr24:22,684,454-22,686,432	CAGGACCCTGAGAAACGAAA	CCAGGAGCGTGTAATGGTCT
lncRNA transcript	chr24:26,327,936-26,328,024	CTGCTGAGCTCTCTGGGTGT	CAGCTGGAGTGACTGAGCATT
SEL1L genotyping	chr24:26,080,981-26,082,166	CAACGTCAGTGCCAAATCC	ACATTGCCTTTCCAGCAGTC
VIPAR genotyping	chr24:22,685,271-22,685,559	TGAGCCACAGCCACTTGTTA	TTCCAGACCCGTCTAGCATC
ACTB transcript	chr13:4,382,919-4,383,011	AAGGAGAAGCTCTGCTATGTCG	GGGCAGCTCGTAGCTCTTC
Human SEL1L-A (M. Cattaneo et al. 2009)	chr14:81,487,426-81,498,033	CTCGCTAACAGGAGGCTCAGTAGTAC	GCCACTGGCATGCATCTGAGC
Mouse SEL1L-A	chr12:91,815,802-91,824,961	GCCGCTGGCGTGCATCTGTGC	CTCCCTAACTGGAGGCTCTGTAGTCC
Horse SEL1L-A	chr24:26,083,393-26,093,784	GCCGCTGGCATGCATCTGTGC	CTCACTGACAGGAGGCTCAGTGGTAC
Zebrafish SEL1L-A	chr20:26,993,491-26,997,762	GTCTCTGACAGGGGGCAGTGCAGTAC	TCCTGTCGCATGCATTTGGGC
Zebrafish SEL1L Genotyping	KO: chr20:26,989,084-26,999,915<br>WT: chr20:26,999,660-26,999,915	KO: GCATGCCAACAATGCTTTT<br>WT: TTAATGCATCCTTGCTGAAAAA	GAGCCTTGACACGTTGGATT

**Supplemental Table 2: Number of variants identified in candidate genes**	
**Gene**	**Total variants**
AKT1	251
AKT2	181
F2	166
F2R	64
F5	448
FGA, FGB	152
PIK3C2A	329
PIK3C2B	421
PIK3C2G	2598
PIK3CA	97
PIK3CB	688
PIK3CG	128
PIK3R1	374
PIK3R4	1144
PIK3R5, PIK3R6	602
SHIP1	721

Supplemental Table 3: Associated Variants with P<0.0001			
**Position**	**P-values**	**Nearest Gene**	**Expression and Interaction in Platelets**
chr24:22162998	3.74E-05	*ANGEL1*	No
chr24:22642450	3.74E-05	*SAMD15*	No
chr24:22642470	3.74E-05	*SAMD15*	No
chr24:22888518	3.74E-05	*ALKBH1*	No
chr24:26081856	7.63E-06	*SEL1L*	Yes
chr24:21867050	3.74E-05	*ESRRB*	No
chr24:21999935	3.74E-05	*ESRRB*	No
chr24:22685398	3.74E-05	*VIPAR*	Yes
chr24:24095086	9.73E-06	*NRXN3*	No
chr24:24460139	9.73E-06	*NRXN3*	No
chr24:24525130	9.73E-06	*NRXN3*	No
chr24:24709312	2.29E-05	*NRXN3*	No
chr24:25392066	3.74E-05	*CEP128*	No
chr24:25429259	3.74E-05	*CEP128*	No
chr24:26447375	7.63E-06	*SEL1L (AL355838.1)*	Yes

Supplemental Table 4: Cross-species comparisons and similarities		
**Corroborative observations**		**Findings**
TBT (horse)	Tail bleed (mouse)	Lack of functional impact of SEL1L deficiency on minor injury-induced clotting
Collagen spreading (horse)	Collagen flow chamber (mouse)	Reduction in adherence to collagen in the context of SEL1L deficiency
Collagen flow chamber (mouse)	Thrombocyte attachment (zebrafish)	Reduction in adherence of platelets/thrombocytes under flow in SEL1L deficiency
Megakaryocyte expression (human)	Megakaryocyte expression (mouse)	Demonstrates SEL1L expression in megakaryocytes across development
RT-PCR for *SEL1L-A* in platelets/thrombocytes (human, horse, mouse, zebrafish)		Demonstrates *SEL1L* is present in platelets/thrombocytes in all species studied
