# Supplementary material for: ER-associated degradation pathway protein SEL1L plays an evolutionarily conserved role in platelet adhesion
Source: J Clin Invest. 2026 Feb 16;136(4):e191433. doi: 10.1172/JCI191433 (PMC12904699; doi:10.1172/JCI191433)

Full unedited blot for FIGURE 5C - MEGAKARYOPOIESIS

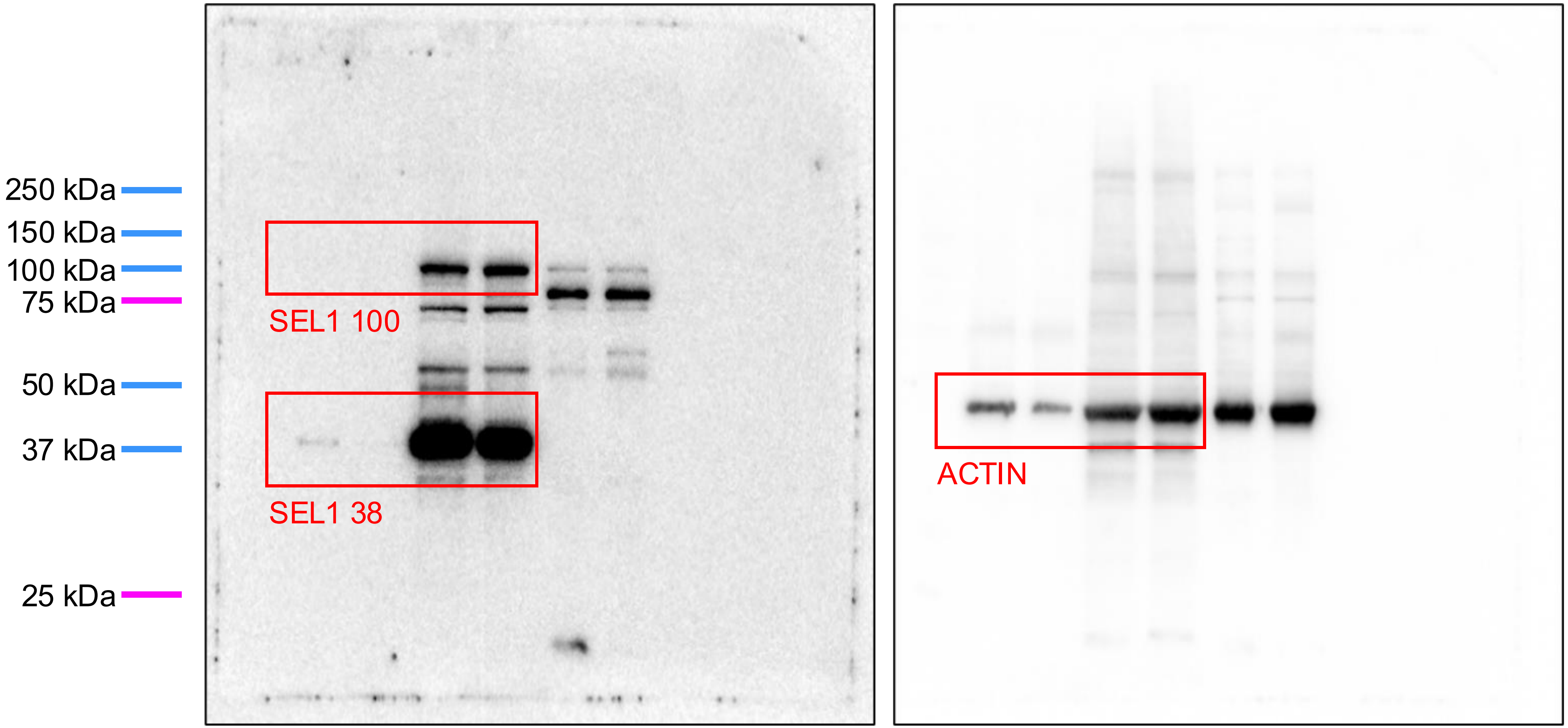

Full unedited blot for FIGURE 5C - PLATELETS

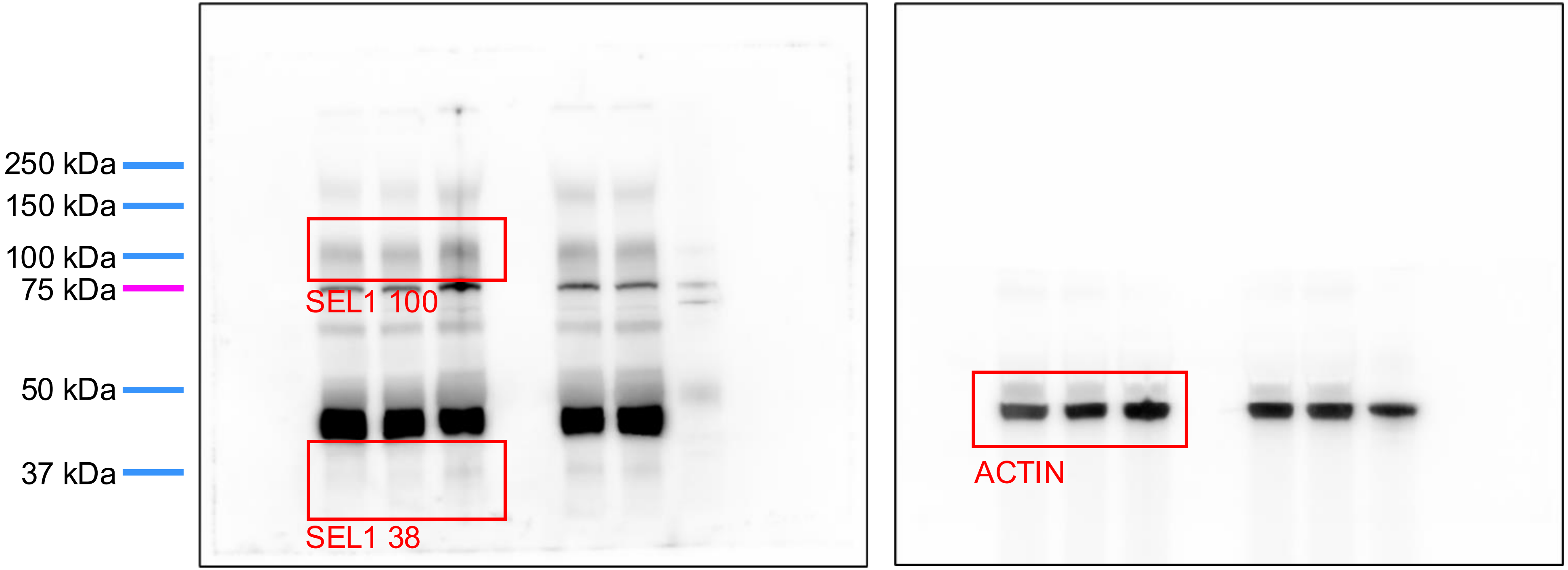

Full unedited blot for SUPPLEMENTAL FIGURE 8C

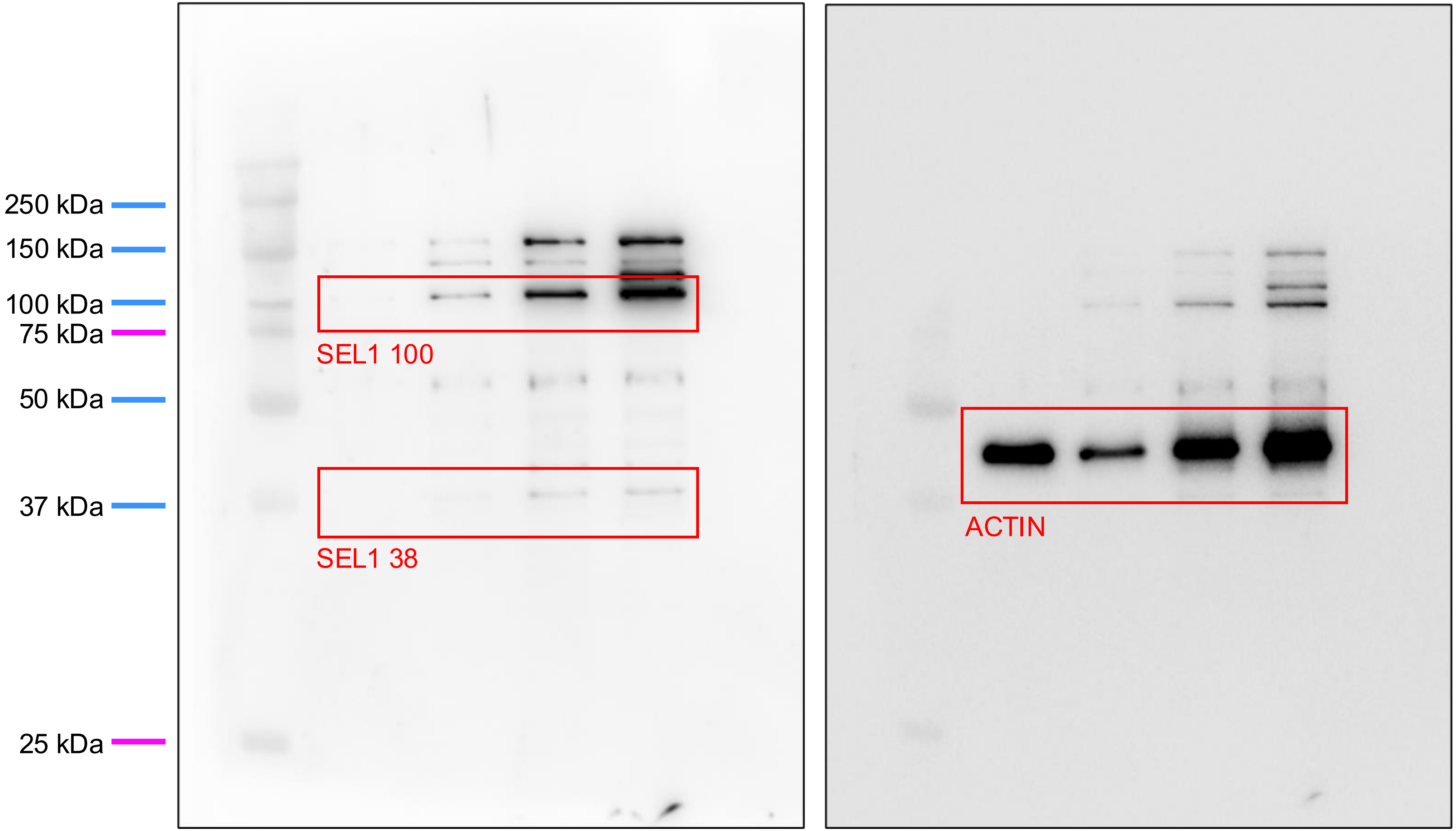

Supplement: Unedited blot and gel images [file jci-136-191433-s165.pdf]
